# Multi-generational benefits of genetic rescue

**DOI:** 10.1038/s41598-024-67033-6

**Published:** 2024-07-30

**Authors:** Dave P. Onorato, Mark W. Cunningham, Mark Lotz, Marc Criffield, David Shindle, Annette Johnson, Bambi C. F. Clemons, Colin P. Shea, Melody E. Roelke-Parker, Warren E. Johnson, Brett T. McClintock, Kristine L. Pilgrim, Michael K. Schwartz, Madan K. Oli

**Affiliations:** 1 Fish and Wildlife Research Institute, Florida Fish and Wildlife Conservation Commission, 298 Sabal Palm Rd, Naples, FL 34114 USA; 2 Fish and Wildlife Research Institute, Florida Fish and Wildlife Conservation Commission, Gainesville, FL 32601 USA; 3 Division of Habitat and Species Conservation, Florida Fish and Wildlife Conservation Commission, Naples, FL 34114 USA; 4 US Fish and Wildlife Service, Florida Ecological Services Field Office, Immokalee, FL 34142 USA; 5 Big Cypress National Preserve, National Park Service, Ochopee, FL 34141 USA; 6 Fish and Wildlife Research Institute, Florida Fish and Wildlife Conservation Commission, St. Petersburg, FL 33701 USA; 7 Frederick National Laboratory of Cancer Research, Bethesda, MD 20892 USA; 8 Department of Biology, Loyola University of Maryland, Baltimore, MD 21210 USA; 9 Marine Mammal Laboratory, Alaska Fisheries Science Center, National Oceanic and Atmospheric Administration, National Marine Fisheries Service, Seattle, WA 98115 USA; 10 USDA Forest Service, National Genomics Center for Wildlife and Fish Conservation, Missoula, MT 59801 USA; 11 Department of Wildlife Ecology and Conservation, University of Florida, Gainesville, FL 32611 USA

**Keywords:** Endangered species conservation, Fitness, Florida panther, Genetic rescue, Inbreeding depression, *Puma concolor*, Inbreeding, Population genetics, Conservation biology, Molecular ecology

## Abstract

Genetic rescue—an increase in population fitness following the introduction of new alleles—has been proven to ameliorate inbreeding depression in small, isolated populations, yet is rarely applied as a conservation tool. A lingering question regarding genetic rescue in wildlife conservation is how long beneficial effects persist in admixed populations. Using data collected over 40 years from 1192 endangered Florida panthers (*Puma concolor coryi*) across nine generations, we show that the experimental genetic rescue implemented in 1995—via the release of eight female pumas from Texas—alleviated morphological, genetic, and demographic correlates of inbreeding depression, subsequently preventing extirpation of the population. We present unequivocal evidence, for the first time in any terrestrial vertebrate, that genetic and phenotypic benefits of genetic rescue remain in this population after five generations of admixture, which helped increase panther abundance (> fivefold) and genetic effective population size (> 20-fold). Additionally, even with extensive admixture, microsatellite allele frequencies in the population continue to support the distinctness of Florida panthers from other North American puma populations, including Texas. Although threats including habitat loss, human-wildlife conflict, and infectious diseases are challenges to many imperiled populations, our results suggest genetic rescue can serve as an effective, multi-generational tool for conservation of small, isolated populations facing extinction from inbreeding.

## Introduction

The long-term persistence of imperiled species in the wild is increasingly hampered by numerous challenges worldwide^1,2^. Isolation and small population size, often catalyzed by anthropogenic stressors, can adversely impact population persistence. Inbreeding depression, a reduction in fitness resulting from mating among related individuals, is a conservation challenge common to imperiled populations. The synergy between small population size, isolation from conspecific populations, inbreeding depression, as well as demographic and genetic stochasticity, often results in an “extinction vortex” that can ultimately lead to extirpation of species or populations^3^.

Genetic rescue–an increase in population fitness following the introduction of new alleles–has been a rarely applied, yet successful management initiative for several taxa that were negatively affected by small population size, severe bottlenecks, and geographical isolation^4^. For example, genetic rescue has improved demographic performance and prevented potential extinction of wolves (*Canis lupus*) in Scandinavia^5^, prairie chickens (*Tympanuchus cupido*) in Illinois, USA^6,7^, and wood rats (*Neotoma magister*) in Indiana, USA^8^. The endangered Florida panther (*Puma concolor coryi*) population faced similar challenges through most of the twentieth century^9^. Habitat loss and the impacts of unregulated take combined to reduce the population to approximately 20–30 individuals isolated at the southern end of peninsular Florida by the mid-1990s, > 1000 km from the nearest population of conspecifics^10,11^. Early studies (1981–1995) conducted by the Florida Game and Fresh Water Fish Commission (now Florida Fish and Wildlife Conservation Commission [FWC]) revealed that the panther population was characterized by low levels of genetic variation and suffered from high frequencies of phenotypic (e.g., midline dorsal cowlick of fur, kinked tails) and congenital abnormalities (e.g., atrial septal defects [ASD], cryptorchidism^12,13^) thought to be indicative of inbreeding depression. These abnormalities, combined with poor demographic performance of the population, portended the imminent extinction of the panther and spurred wildlife managers to implement a plan for genetic rescue^14^ centered on the release of 8 female pumas from Texas (*P.c. stanleyana*) into South Florida in 1995. Initial assessments revealed numerous fitness benefits to panthers^15–18^, most notably an increase in the minimum population size to 119 adults and subadults by 2015^10,19^. The continued collection of this multi-generational data set from a wild population of large carnivores during more than 4 decades of research is unprecedented and sets the stage for a unique opportunity to track long-term consequences of genetic rescue. This knowledge could prove beneficial to the conservation of other imperiled wildlife beyond apex predators.

Although genetic rescue has been successfully applied as an effective management tool^4,20^, the persistence of benefits accumulated from the initial influx of genetic variation into the population remains largely unknown^21–23^. Some studies have addressed this question experimentally^24,25^, but few have evaluated the long-term effects of genetic rescue within a wild population beyond the F2 generation^4^. We would expect “hybrid vigor”^26^ to be associated with the F1 admixed individuals because of heterosis and subsequent improvements to population fitness incurred from genetic rescue^4^. The benefits of genetic rescue beyond the F1 generation, however, are still debated. A meta-analysis incorporating data from 156 studies and 77 species supported the benefits of outcrossing experiments often lasting beyond the F3 generation^27,28^. However, > 80% of the data sets analyzed by Frankham^27,28^ involved short-lived invertebrates or plants, and only 30 of 156 studies examined fitness beyond F2, of which just one involved a vertebrate species (desert topminnow, *Poeciliopsis monacha*).

Here, we used morphological, biomedical, demographic, and genetic data collected from 1192 Florida panthers over 40 years—inclusive of data through the F5 generation (generation time = 4.45 years^16^) following a single genetic rescue event in 1995—to provide an unprecedented assessment of the persistence of benefits to the population. We examined morphological and biomedical correlates of inbreeding, genetic variation, population genetic structure, inbreeding coefficients, estimates of abundance prior to the implementation of genetic rescue in 1995, and generational changes in these characteristics post-genetic rescue. Lastly, we present the most comprehensive estimate of genetic effective population size^29^ for Florida panthers to date, highlighting improvements following genetic rescue. Our findings clearly demonstrate the phenotypic, genetic, and demographic benefits of genetic rescue and track how these improvements persist temporally across five generations, both critical components for assessing genetic rescue as a tool for long-term management of small, isolated, and inbred populations worldwide.

## Results

### Sampling, microsatellite genotyping and genetic ancestry

**Figure 1:**
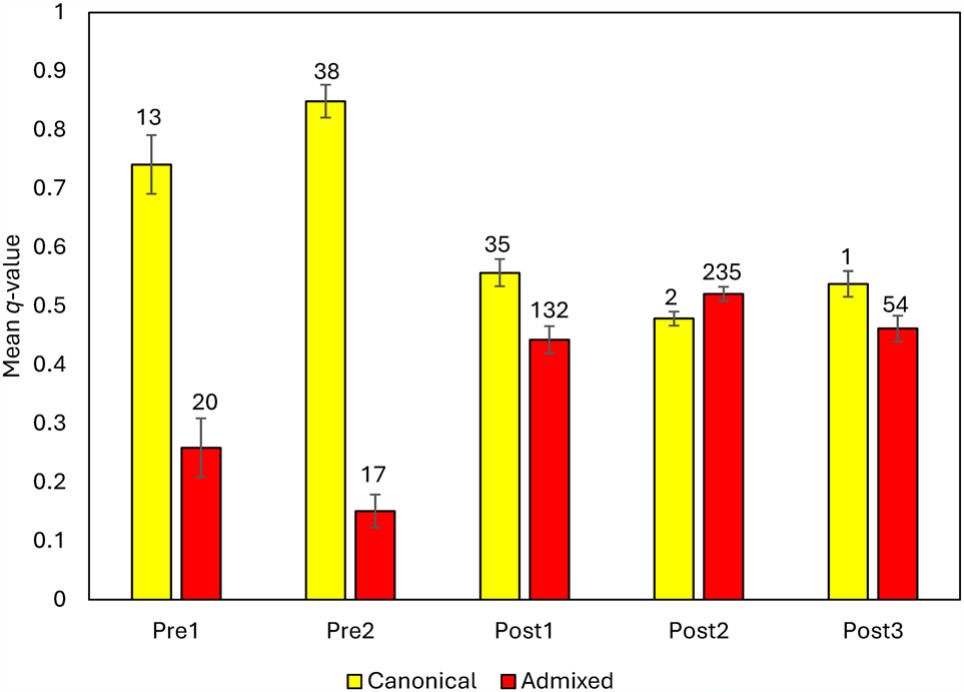
Ancestry of adult and subadult Florida panthers (n = 547) sampled 1981–2020 in Florida, USA. Ancestry was determined via a clustering analysis of microsatellite genotype data using the program STRUCTURE with *K* = 2 clusters (canonical and admixed). Cohorts include Florida panthers born during the pre-genetic rescue (Pre1 and Pre2) and post-genetic rescue (Post1–3) periods (see “Methods”). Panthers were designated as canonical if their canonical ancestry *q*-value was ≥ 90%. Mean* q*-values for each ancestry category are presented to demonstrate changes in the composition of population ancestry across generations of panthers pre- and post-genetic rescue. The SE values are reflective of the combined sample within each cohort, since *q*-values presented are proportions of that sample. Values above each bar represent the number of panthers assigned to that ancestral category in the pre- or post-genetic rescue cohort.

From 1981 to 2021, we examined 1192 Florida panthers (dependent-aged kittens, subadults, and adults) to collect tissue samples and to document biomedical and morphological abnormalities. Tissue samples collected from Florida panthers were used to obtain genotypes at 16 microsatellite loci; this dataset encompassed 9 panther generations, including 4 generations prior to and 5 generations after genetic rescue (descriptive statistics on microsatellite loci are provided in *Supplemental Information [SI] *Appendix 1). Our analysis of genetic ancestry using a Bayesian clustering algorithm in program STRUCTURE^30^—with genotype data from 904 panthers, 49 Texas pumas (including 7 of the 8 Texas female pumas introduced in 1995 [TX102 did not amplify]), and 12 additional non-Florida pumas—supported a two-cluster model (Δ*K* = 1786 at *K* = 2, Ln likelihood _(K=2)_ = − 31,951, SD = 0.8). From this model, we used *q*-values (proportion of an individual’s genome [i.e., ancestry] originating from a cluster in *K*) to assign panthers as either canonical (≥ 90% pre-introgression ancestry) or admixed (< 90% pre-introgression ancestry) for subsequent analyses (*SI *Appendix 2). The increased level of admixture in cohorts of panthers born post-genetic rescue (Post 1–3 cohorts; see “Methods”) is apparent when compared to the pre-genetic rescue cohorts (Pre1–2; Extended Data Fig. 1; *SI *Appendix 2). Using a subset of 547 adult and subadult panthers, the mean *q*-value for the cluster associated with canonical panthers was at its peak in the cohort just prior to genetic rescue (Fig. 1; Pre2; mean canonical ancestry of *q* = 0.849 SE = 0.028, n = 55), but generations of panthers in cohorts following genetic rescue had significant admixture (Fig. 1).

### Morphological and biomedical correlates of inbreeding

**Figure 2:**
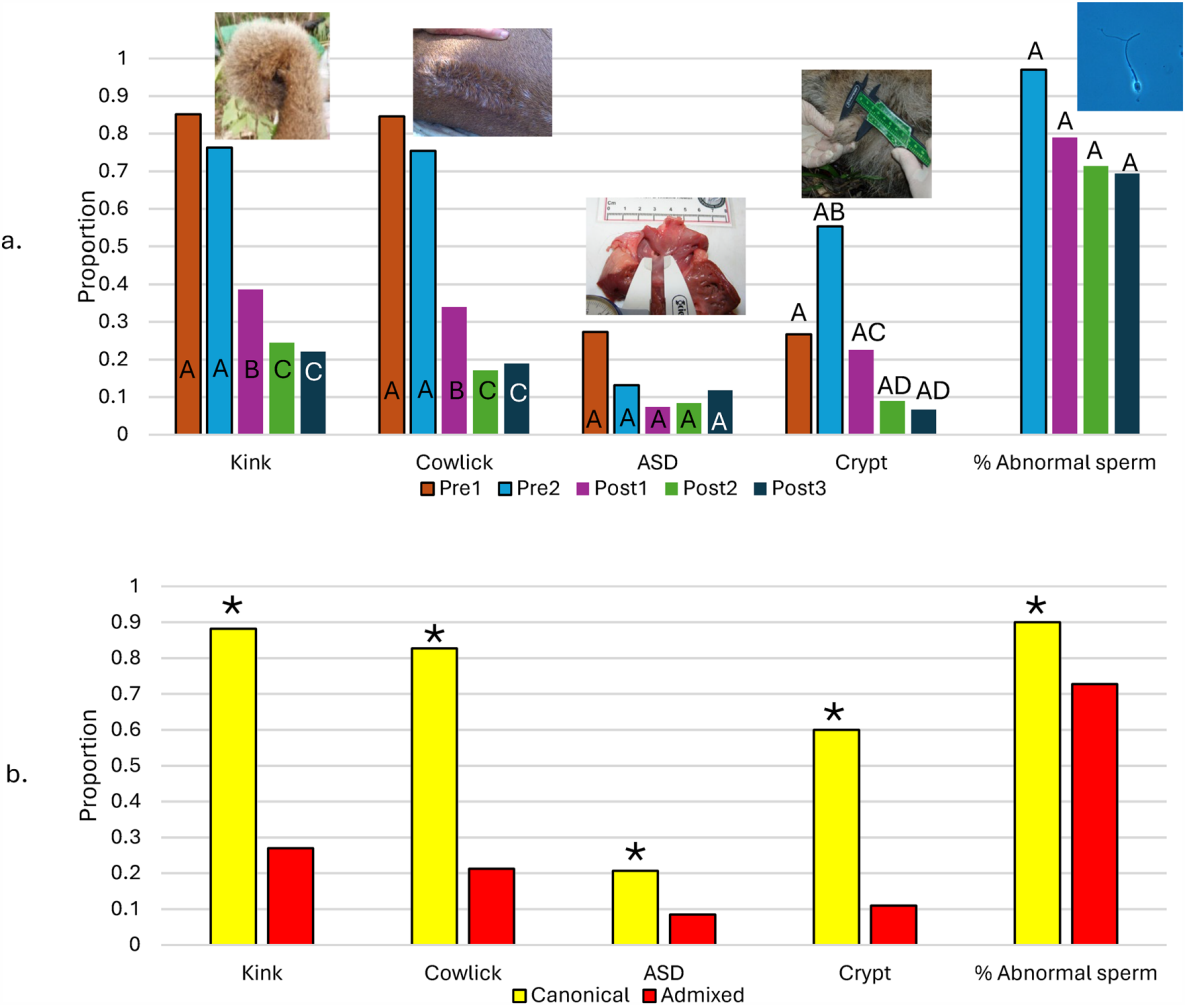
Proportion of Florida panthers sampled from 1981 to 2021 in Florida, USA, that exhibited correlates of inbreeding depression for morphological and physiological traits. Comparisons are made via panthers born in cohorts pre- and post-genetic rescue (**a**) and between defined ancestral categories (**b**). The percent abnormal sperm data are from Penfold et al.^46^. Statistical analyses are detailed in *SI Appendix 3*. Different letters in Frame (**a**) indicate significant differences (*P* < 0.05; binomial regression analyses with post-hoc comparisons) between cohorts of panthers. The Post3 cohort includes data from 2021 for these correlates of inbreeding. We used two proportion Z-tests to determine significant comparisons (*P < 0.05) between ancestral categories on Frame (**b**) (*SI Appendix 3*).  ASD = Atrial Septal Defect and Crypt = Cryptorchidism.

From 1981 to 2021, we examined 612 Florida panthers for morphological and biomedical abnormalities, including the kinked tail, dorsal cowlick of fur, ASDs, cryptorchidism, and percentage of abnormal sperm. Between the pre-genetic rescue and Post3 cohorts, the proportion of panthers with kinked tails (0.852 to 0.221), cowlicks (0.846 to 0.189), and cryptorchidism (0.553 to 0.067) all significantly declined (Fig. 2A; statistical results are presented in *SI *Appendix 3). The proportion of panthers with ASD and the percentage of abnormal sperm in males also substantially declined from pre- to post-genetic rescue periods, but these changes were not statistically significant because of small sample sizes (Fig. 2A; *SI *Appendix 3). Admixed panthers were significantly less likely to express any of the correlates of inbreeding when compared to canonical panthers (Fig. 2B; *SI *Appendix 3).

### Measures of genetic variation

**Figure 3:**
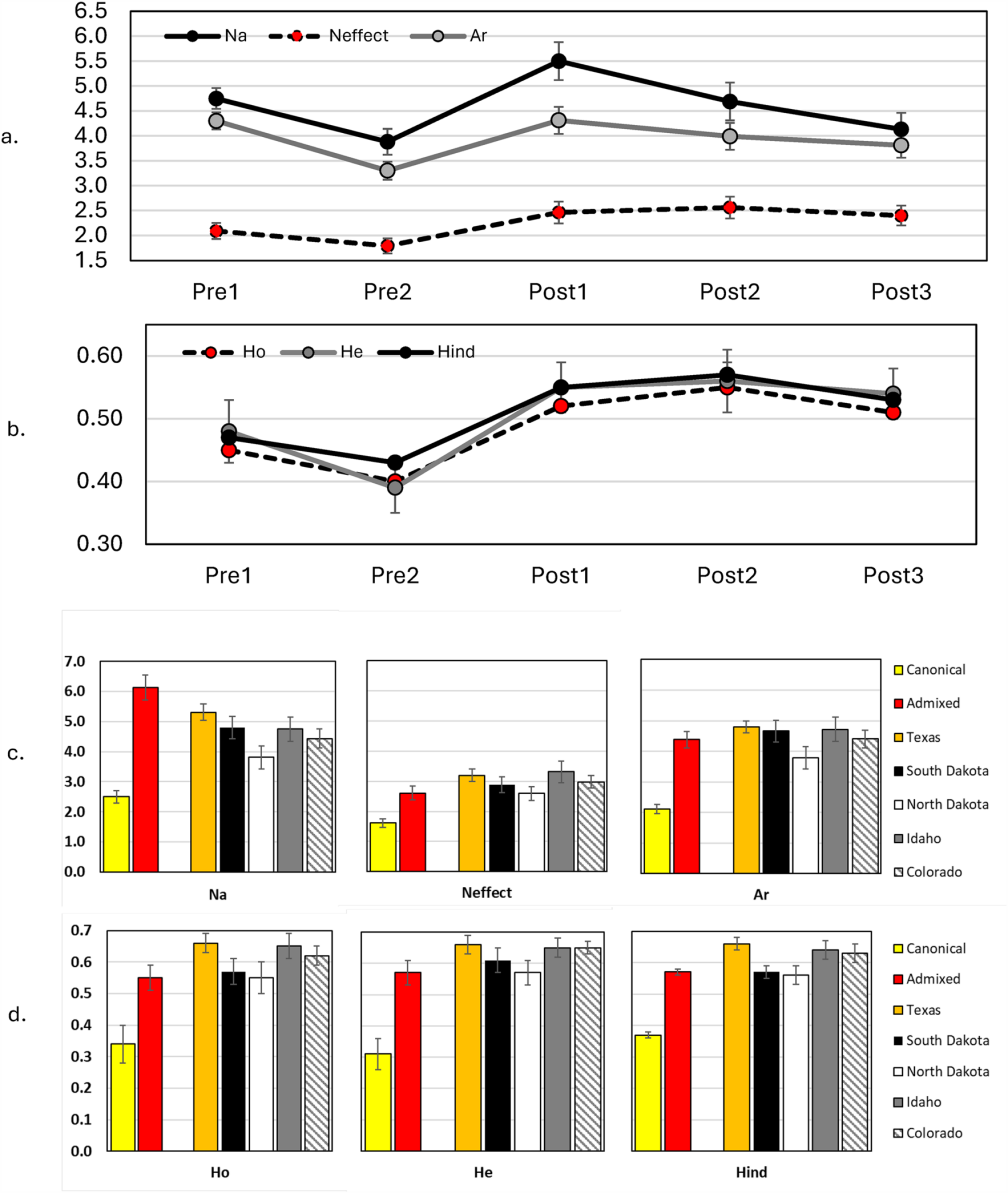
Metrics of genetic variation calculated using genotype data from 16 microsatellite loci in cohorts of Florida panthers pre- and post-genetic rescue (Frames **a** and **b**) and ancestral groups (Frames **c** and **d**) sampled from 1981 to 2020 in Florida, USA. Values are means and standard errors. Data from Western populations of puma are presented for comparative purposes. Metrics include number of alleles (N_a_), number of effective alleles (N_effect_), observed and expected heterozygosity (H_o_, H_e_), allelic richness (A_r_), and individual heterozygosity (H_ind_). Statistical analyses methods and results comparing these groups are presented in *SI Appendix 4*.

We assessed genetic diversity of the panther population based on microsatellite genotypes from 552 tissue samples of adult and subadult Florida panthers (i.e., excluding kittens) collected from 1981 to 2020 (see “Methods”). The Pre2 cohort was characterized by the lowest levels of genetic variation; all measures—including number of alleles (N_a_), number of effective alleles (N_effective_), allelic richness (A_r_), observed (H_o_), expected (H_e_), and individual (H_ind_) heterozygosity—showed substantial improvements following the implementation of genetic rescue and achieved the highest values in the Post1 or Post2 periods (Fig. 3A,B). For example, A_r_ increased from 3.30 for the Pre2 cohort to 4.31 in Post1 cohort, and H_o_ increased from 0.40 during Pre2 to 0.55 during Post2 (Fig. 3A,B; see *SI *Appendix 4 for pairwise contrasts). Overall, post-genetic rescue cohorts of panthers exhibited values of these metrics of genetic variation that were comparable to larger contiguous populations of pumas in the Western United States (Fig. 3A–D; *SI *Appendix 4, Table A4.1). Similarly, admixed panthers were genetically more diverse than canonical panthers across all measures of genetic variation (e.g., H_e_ = 0.31 and 0.57 for canonical and admixed panthers, respectively) and exhibited values more comparable to those of Western puma populations (Fig. 3C,D; *SI *Appendix 4, Table A4.1).

**Figure 4:**
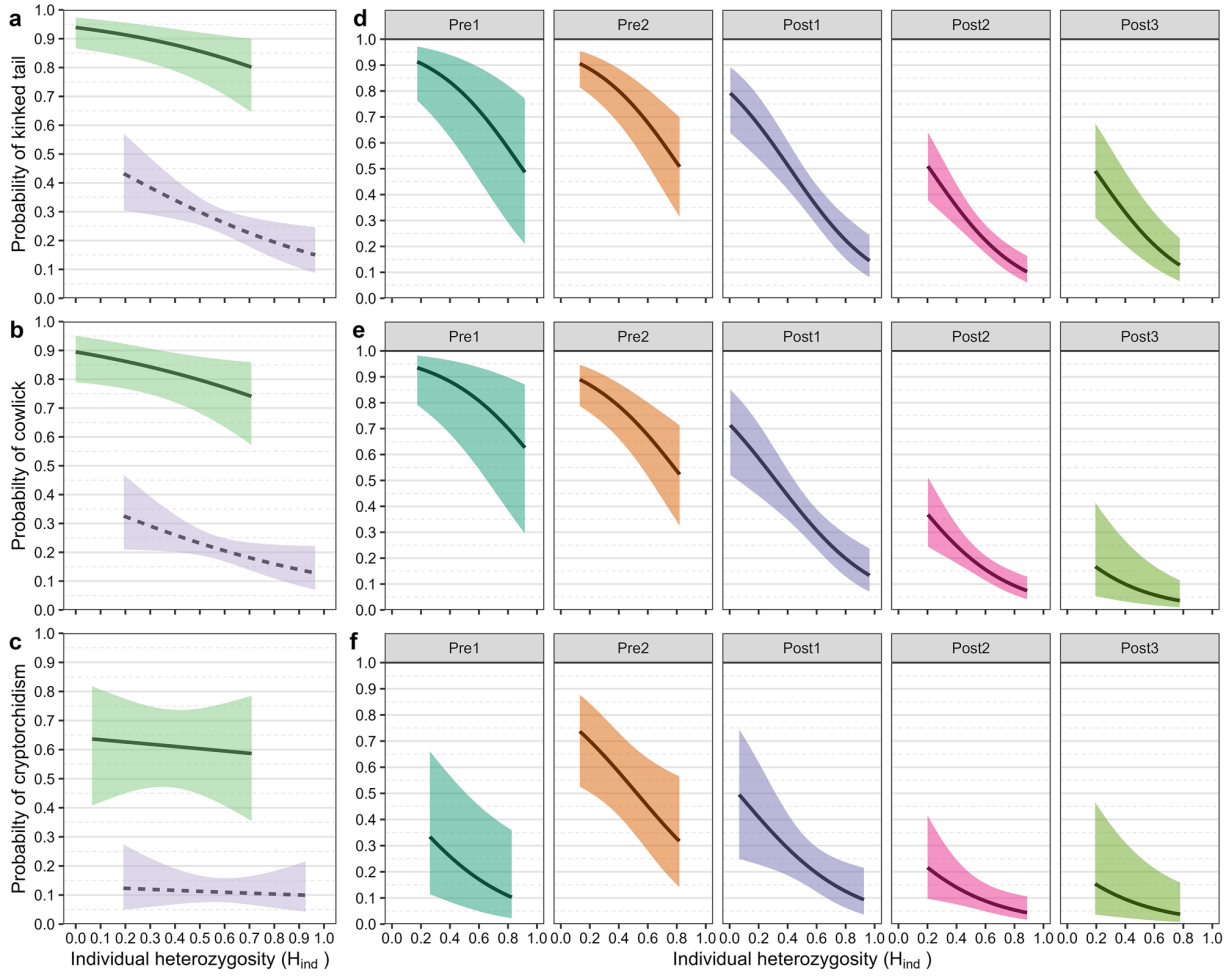
Assessment of the heterozygosity-fitness correlations between individual heterozygosity (H_ind_) and the probability of presence of kinked tails, cowlicks, and cryptorchidism in Florida panthers. Frames (**a**–**c**) depict comparisons of panthers categorized as canonical (solid line) versus admixed (dashed line). Frames (**d**–**f**) represent comparisons between cohorts of panthers from the pre- and post-genetic rescue periods. Shaded areas represent 95% confidence intervals.

Our comparisons of H_ind_ between different cohorts of panthers and between ancestral groups showed significant improvements in the post-genetic rescue era and in admixed panthers (Fig. 3B,D; *SI *Appendix 5). The H_ind_ values for these same groups were statistically similar to those of Western puma populations in most cases, while canonical, Pre1 and Pre2 groups were significantly lower in most comparisons (*SI *Appendix 5, Tables A5.1, A5.2, and Fig. A5.1). Our evaluation of the heterozygosity-fitness correlation (HFC) between H_ind_ and several correlates of inbreeding (kinked tails, cowlicks, and cryptorchidism) showed a substantial reduction in the probability of the expression of those traits in Florida panthers across all cohorts and for both canonical and admixed panthers as H_ind_ increased (Fig. 4).

### Genetically effective population size, abundance and principal coordinate analysis

**Figure 5:**
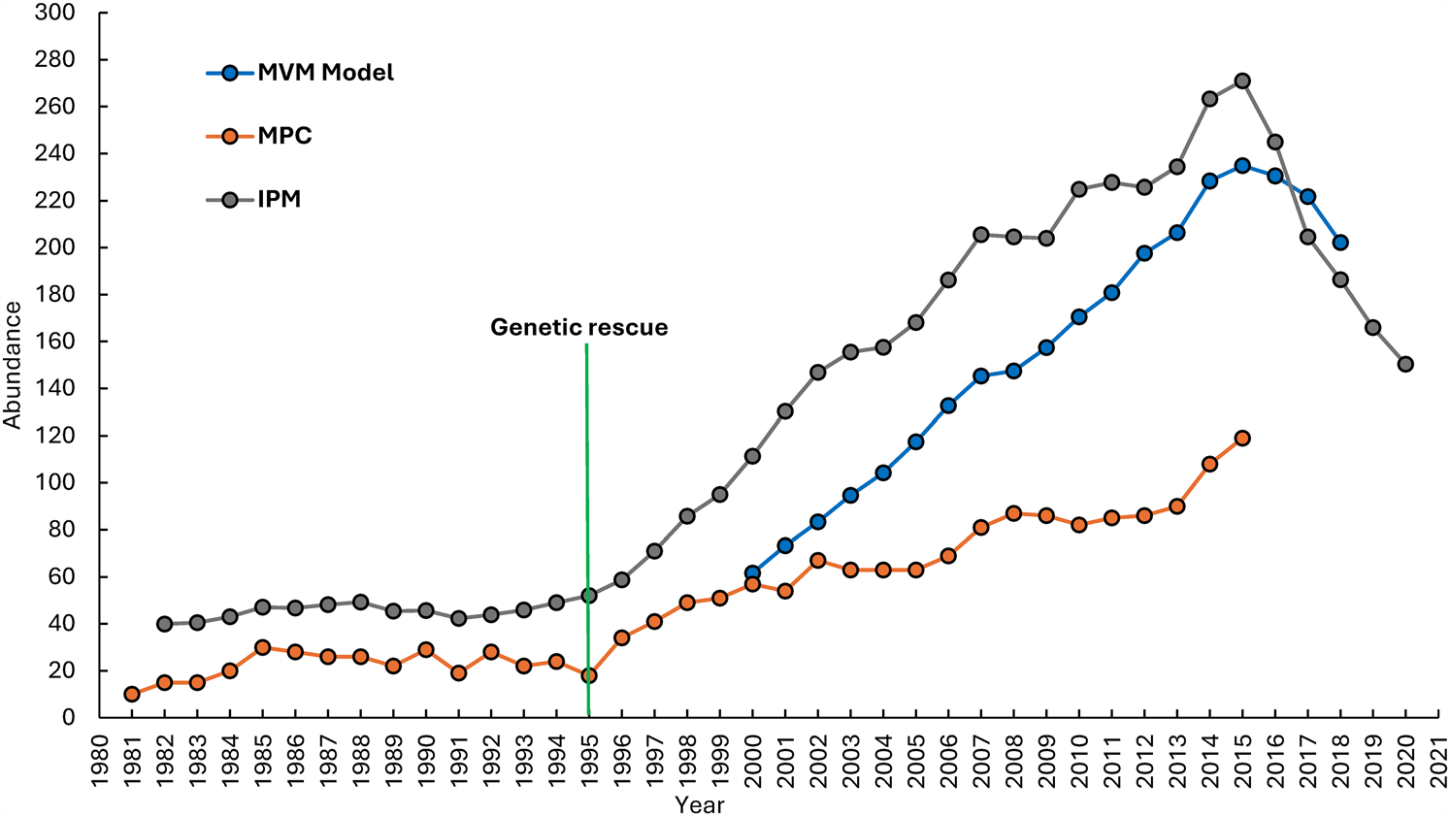
Estimates of the range-wide population size of adult and subadult Florida panthers from 1981 to 2020 using: (1) the 95% lower confidence interval of the motor vehicle mortality (MVM) model-averaged abundance estimate via the method of McClintock et al.^72^ plotted in blue for the period 2000–2018 (see *SI Appendix 6*); (2) the minimum population count (MPC) index of McBride et al.^10^ and McBride and McBride^19^ plotted in orange, for the period 1981–2015; (3) and the integrated population model (IPM) of Merriell^73^ in plotted in grey, for the period 1982–2020. The year in which genetic rescue was initiated (1995) coincides with the subsequent increase in the population size that was documented by all three metrics.

By applying the linkage disequilibrium method^31^ within NeEstimator 2.1^32^ to our microsatellite data, we estimated genetically effective population size (*N*_*e*_) for Florida panthers across a temporal scale and for ancestral groups (Extended Data Fig. 2). Prior to genetic rescue, the panther population was close to extirpation, with an *N*_*e*_ = 2.7 (95% CI 2.4–3.1). Following genetic rescue, *N*_*e*_ increased consistently, reaching a value of 62.1 (95% CI 40.2–115.5) during the Post3 period (Extended Data Fig. 2). Admixed panthers had substantially larger *N*_*e*_ than canonical panthers (Extended Data Fig. 2). The observed increase in *N*_*e*_ mirrors the documented population growth in our index and estimates of Florida panther abundance (Fig. 5; *SI *Appendix 6).

**Figure 6:**
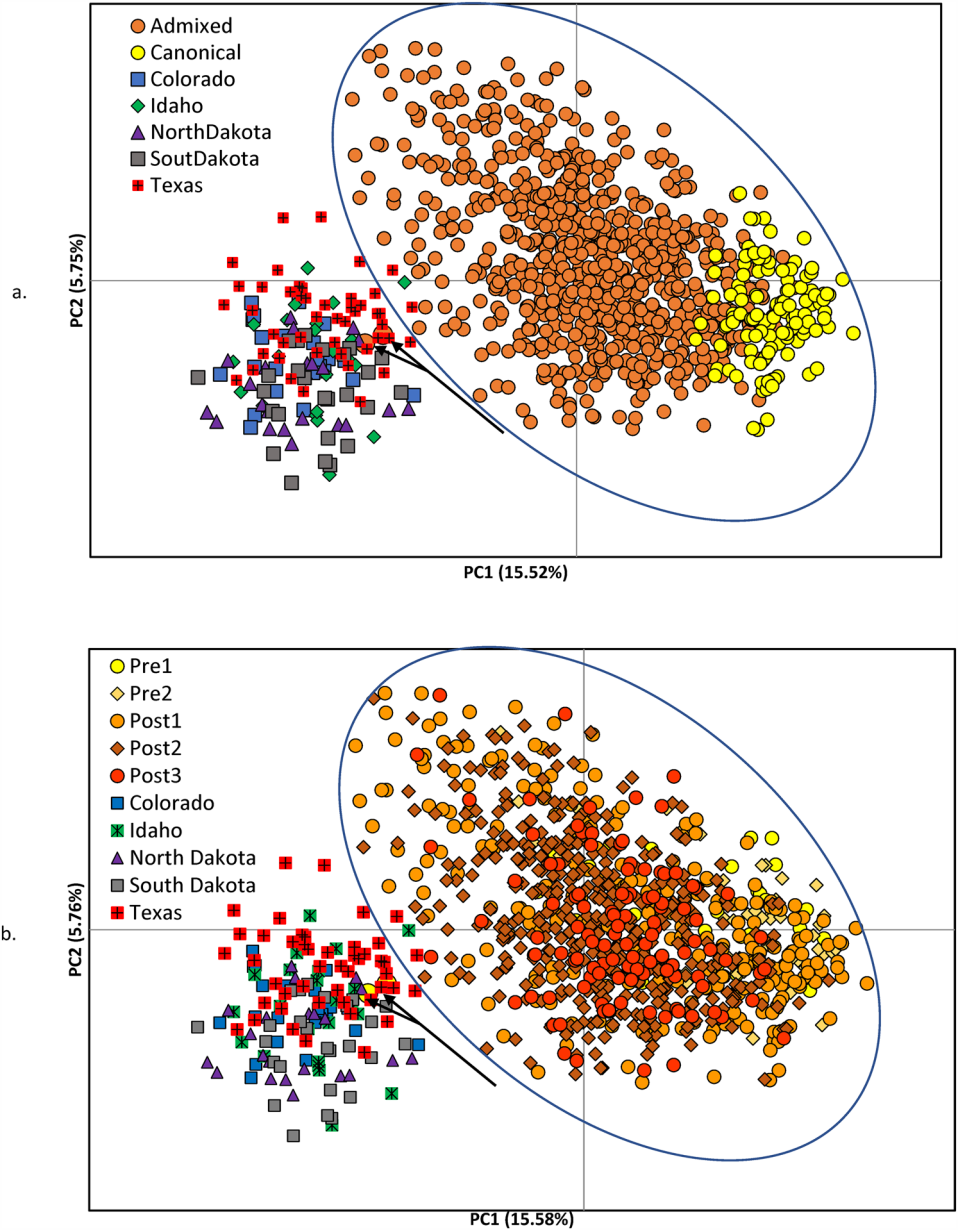
Principal coordinate analysis (PCoA) based on Nei’s genetic distance matrices between (**a**) canonical and admixed Florida panthers and Western puma populations, and (**b**) Pre1, Pre2, Post1, Post2, and Post3 Florida panthers and Western puma populations. Ovals highlight the distinction of the cluster of Florida panther samples from the Western puma populations, including Texas. The arrows point to two uncollared Florida panther samples (UCFP010 and UCFP011; circles enlarged for clarity) collected in Palm Beach County Florida in 1983 and 1984, respectively. Both were suspected of being captive pumas that had been released or escaped into the wild. Those suspicions are further corroborated by our genetic analyses and the clustering of these two samples with non-Florida panthers. Variance explained by each PCoA axis is displayed in axis titles.

Our principal coordinate analysis (PCoA) completed in GenAlEx 6.5^33,34^ illustrates the genotypic differences between Western puma populations and panthers via ancestry and cohorts through time pre- and post-genetic rescue. The PCoA shows the historic and continued separation between panthers from Florida and Western puma populations, including Texas, even five generations post-genetic rescue (Fig. 6).

## Discussion

Although initially controversial, the choice to embark on the genetic rescue of the Florida panther was a timely and wise decision that had an immediate and long-term benefit to the population. Virtually all morphological, genetic, and demographic parameters improved following this management initiative. Furthermore, our findings demonstrate the persistence of these benefits through the F_5_ generation post-genetic rescue, a significant improvement in population abundance and N_e_, as well as continued genetic distinctness of the admixed panthers from Western puma populations.

A goal of the genetic rescue plan was a 20% level of introgression of the Texas puma genome into the Florida population^14^. This was quickly surpassed, as evidenced by the 44% mean admixed ancestry immediately following genetic rescue in the Post1 (1996–2005) cohort of panthers (Fig. 1; *SI *Appendix 2, Table A2.1). An admixture level of 44–52% in post-genetic rescue cohorts is more than twice the level deemed adequate to forestall inbreeding^14^. High levels of admixture following genetic rescue can potentially result in outbreeding depression—a swamping of ancestral, locally adapted, beneficial traits in a population^35^—and was cited as a possible concern for the F1 and F2 generations of panthers^36^. Nevertheless, there are alternative perspectives on the levels of admixture required for successful genetic rescue attempts that avoid outbreeding depression. For instance, Harris et al.^37^ demonstrated through model simulations how inbred Neanderthals (*Homo neandertalensis*) benefited genetically when introgressed with modern humans (*H. sapiens*), even with the loss of a significant portion of their native ancestry. Fitzpatrick et al.^38^ showed that genetic rescue in Trinidadian guppy (*Poecilia reticulata*) populations did not result in swamping of local adaptation but instead produced hybrids (70–80% admixed) with improved levels of fitness as well as a concomitant increase in population size post-genetic rescue. Overall, examples of outbreeding depression in mammals are rare, especially when contrasted with the well documented negative effects of inbreeding depression within imperiled populations^39^. To date, we have not identified evidence of outbreeding depression in the panther population.

The kinked tail, cowlick, cryptorchidism, and ASDs have become infamous traits of the Florida panther population and manifestations of inbreeding depression^13,40, 41^ that have been used in other wildlife populations to identify the potential presence of inbreeding^42,43^. Our findings that morphological and biomedical correlates of inbreeding consistently declined from the pre- to post-genetic rescue cohorts provide clear evidence of the immediate and long-term benefits of genetic rescue. For example, kinked tails and cowlicks were documented in > 75% of panthers sampled prior to genetic rescue but were found in fewer than 30% of panthers sampled post-genetic rescue. We note, however, that caution must be exercised when interpreting levels of inbreeding depression based only on phenotypic indicators of inbreeding (e.g., kinks and cowlicks); direct measures of fitness or its components, such as life history parameters, are preferred when available^44^. That said, our previous research on panthers correlating metrics associated with inbreeding depression (e.g., heterozygosity, percent abnormal sperm) with direct traits of fitness such as survival^17,45^ and improved prospects for reproductive success^46^ supports the use of indirect traits easily identified via routine examination as proxies to monitor the prevalence of inbreeding in this population.

When assessing measures of genetic variation within cohorts of panthers born pre- or post-genetic rescue, the pattern was a consistent decline in all variables from Pre1 to Pre2 and then improvements to post-genetic rescue cohorts (Fig. 3A,B; pairwise contrasts results appear in *SI *Appendix 4). Comparisons between these cohorts and Western pumas typically followed a trend of lower values for Pre1 and Pre2, while most contrasts with post-genetic rescue cohorts were not different (*SI *Appendix 4). Focusing on H_e_ reveals clear improvements accrued to the population during the post-genetic rescue period, with the highest level observed four generations after genetic rescue (0.56 ± 0.04 for Post2). This level of H_e_ approaches those calculated for Western pumas in our study (*SI *Appendix 4, Table A4.1) and large contiguous populations in California and New Mexico^47,48^.

All measures of genetic variation revealed significant improvements when comparing canonical versus admixed panthers (Fig. 3C,D). Additionally, admixed panthers exhibited values for these metrics of genetic variation that were comparable to those of Western puma populations (Fig. 3C,D; *SI *Appendix 4, Table A4.1). For example, H_e_ was 0.31 ± 0.05 for canonical panthers versus 0.57 ± 0.04 for admixed panthers, which was similar to H_e_ for Western pumas (range: 0.57–0.66), highlighting the improved level of genetic diversity in admixed panthers. A puma population in the Santa Ana Mountains that is isolated between the metropolises of Los Angeles and San Diego, California, had an H_e_ of 0.33^47^, which was almost as low as that for canonical panthers. Gene flow to this California population requires that periodic migrants access the Santa Ana Mountains from nearby ranges that encompass a larger metapopulation of pumas, a herculean challenge given extensive development and highways separating the populations^49^. Unfortunately, Florida panthers do not have a population of conspecifics within dispersal range, which impairs the population’s ability to maintain genetic diversity and reduce genetic drift.

Inbreeding depression in wild populations can be evaluated using heterozygosity–fitness correlations (HFC)^50,51^. Results of our comparisons of H_ind_ values resembled trends for other metrics of genetic variation in the population, with canonical panthers having significantly lower H_ind_ compared to admixed and all Western pumas (*SI *Appendix 5, Table A5.1 and Fig. A5.1). Our assessment of the HFC associated with H_ind_ and the probability of expressing several correlates of inbreeding (kinks, cowlicks, cryptorchidism) revealed that canonical panthers had a higher probability of expressing traits compared to admixed panthers (Fig. 4A–C), and pre-genetic rescue cohorts of panthers had a higher probability of expressing these traits than post-genetic rescue cohorts (Fig. 4D–F). Most importantly, regardless of ancestry or cohort, an increase in H_ind_ coincided with a decrease in the probability of expressing one of these correlates of inbreeding.

Historically, much of the research on HFCs has focused on populations that were not threatened or endangered^52^, yet these findings were often touted as being applicable to species of conservation concern. We identified an HFC in an imperiled population, and our results support the premise that increased H_ind_ levels are associated with a reduction in the prevalence of inbreeding correlates. Comparing results for canonical and admixed panthers allowed us to assess how HFCs are affected in a population that has undergone a severe bottleneck versus an outbred population. Previous research corroborated several HFCs in the panther population, including the positive impacts of increased heterozygosity on survival, male reproductive parameters, and population viability analyses^16,17, 45, 46^. These results support the use of HFCs to identify inbreeding depression, something that has been suggested for other endangered mammals, such as the Iberian lynx (*Lynx pardinus*) and mhorr gazelle (*Nanger dama mhorr*)^50^.

Our analyses of population abundance and genetic effective population size (N_e_) revealed just how close Florida panthers came to extinction during the 1980s and early 1990s. Prior to genetic rescue in 1995, the panther population numbered just 10–49 individuals (Fig. 5) and had a critically low N_e_ of 2.7–3.2 panthers (Extended Data Fig. 2). This range for N_e_ for the cohorts of predominantly canonical panthers is comparable to the only other estimate of N_e_ based on a small data set (13 specimens; Ref.^11^). Culver et al.’s^11^ analyses of nuclear and mitochondrial DNA from historic (< 1922) and contemporary (1980s) canonical Florida panther samples revealed that the population may have endured a bottleneck where it was as small as only 6 individuals (N_e_ = 2) at one point last century. Our Pre1 and Pre2 cohorts (77 samples), with some specimens dating back to the 1970s, further corroborate our observation that panthers were threatened by extirpation. The cohorts sampled following the genetic rescue (Post1, Post2, and Post3) exhibited a sustained increase in N_e_, which was also reflected in the index and estimates of abundance that increased > fivefold from pre-genetic rescue levels to > 200 panthers (Fig. 5). The most recent cohort (Post3) has an estimated N_e_ of 62.1 (parametric 95% CI 40.2–115.5) panthers, a remarkable 23-fold increase in N_e_ and a value comparable to those we calculated for several of the larger, Western puma populations (Extended Data Fig. 2). The observed increases in population abundance and N_e_ following genetic rescue are consistent with other improvements we have documented in the population, including improved demographic performance and substantial reduction in the probability of extinction^16,45^.

Our PCoA of genotype data collected over 40 years demonstrated the continued separation of Florida panthers and western pumas—including Texas—regardless of ancestry or cohort (Fig. 6A,B). Although admixed panthers since genetic rescue have shifted the cluster of panthers toward Western pumas, this analysis still permits the identification of the geographical origin of a puma DNA sample as from a Florida or non-Florida population. These findings are further supported by: (1) our estimates of genetic structure via *F*_*st*_ between these groups that show low levels of differentiation (0.015–0.071) between all panther cohorts and ancestry groups but elevated levels of differentiation (0.105–0.316) when compared to Western pumas (*SI *Appendix 7, Tables A7.1 and A7.2); and (2) the ancestral cluster from our STRUCTURE *K* = 3 analysis that is dominant in all Western puma populations we sampled (*q*-value $\overline{\text{X }}\text{=0.951}$± 0.008, N = 143) is found at very low percentages in panthers (*q-*value $\overline{\text{X }}\text{= 0.026}$± 0.003, N = 545; see Extended Data Fig. 3; *SI *Appendix 2), regardless of whether they were within pre- or post-genetic rescue cohorts. These data support the distinct genetic signature of Florida panthers that remains after 25 years of admixture and continues to differentiate them from other puma populations in North America.

The evidence is clear that genetic rescue has benefited one of the world’s most endangered populations of large carnivores. Data presented here, whether from correlates of inbreeding, genetic variation, population abundance, or effective population size, show that genetic rescue has alleviated the immediate risk of population extinction and reduced the effects of inbreeding depression beyond the F1 generation of admixed panthers. In fact, our data confirm the improvements to this population through the F5 generation post-genetic rescue (18–22 years). However, panther population abundance has stabilized and declined in recent years (2016–2020; Fig. 5), and all measures of genetic variation slightly decreased in the most recent cohort of panthers (Post3; Fig. 3A,B) inclusive of the 5^th^ and 6^th^ generations post-genetic rescue. The continued isolation of this population from conspecifics ultimately means that additional genetic management will be necessary^14,45^. Population modeling by van de Kerk et al.^45^ recommended that genetic rescue be implemented every 20–40 years with 5–10 individuals from other puma populations. Although wildlife managers continue to monitor the genetic health of panthers 29 years after genetic rescue, these findings suggest the need to consider future genetic management of this population if the most recent trends continue (e.g., declining road mortality, increases in correlates of inbreeding, decreases in abundance estimates).

Similar to other large carnivores worldwide^53,54^, Florida panthers continue to be affected by many anthropogenic stressors including habitat loss and other consequences of increased human encroachment^9^. As of 2020, Florida had over 21 million residents, a 14.6% increase from 2010 (U.S. Census Data Website 2021). Nonetheless, the recent passing of the Florida Wildlife Corridor Act (Florida Statute 259.1055 2021), designed to preserve key wildlife habitat as greenways for species movement in peninsular Florida, may help facilitate northward expansion of the breeding population within its historic range.

Our unprecedented, long-term dataset filled important gaps in our understanding of genetic rescue as a tool for wildlife conservation, specifically the persistence of benefits to a population. There is continued debate on whether there should be a paradigm shift toward increased implementation of genetic rescue in at risk populations^55^ or further development of best practices for monitoring genetic rescue attempts prior to increasing their use^56^. Our findings offer novel insights by showing that the beneficial effects of genetic rescue can persist in the population for at least 5 generations, thereby supporting the implementation of this effective, multi-generational management tool for the conservation and recovery of other small, inbred, and imperiled populations.

## Methods

### Field methods and sampling

Staff from the FWC and National Park Service captured, sampled, radiocollared, and tracked panthers in South Florida from 1981 to 2021 as described by van de Kerk et al.^45^. Information regarding the study area, captures, immobilization, handling, and sampling methods for panther adults, subadults, and kittens are described elsewhere^45,57^. We collected two 4-mm skin biopsies for DNA analysis from the external pinnae of each panther (2 mm from kittens) and stored them in a 2-ml vial with 100% EtOH. In some cases, whole blood collected at capture or tissues collected at necropsy were used for DNA analyses. Plucked hair from the abdomen of panthers served as a backup source of DNA. All live-capture and handling activities were completed under safe and humane protocols approved by the FWC and followed American Society of Mammalogists guidelines^58^.

### DNA extraction and microsatellite genotyping

DNA samples were processed at the National Genomics Center for Wildlife and Fish Conservation (Missoula, MT, USA). Whole genomic DNA was extracted from blood and tissue samples using the Qiagen DNeasy Blood and Tissue kit (Qiagen, Valencia, CA, USA) or via a slight modification (incubation of sample overnight at 60°C on a rocker in a solution containing proteinase K and lysis buffer) of the Qiagen DNA extraction protocol to extract DNA from hair. We used 16 microsatellite loci (Fca090, Fca133, Fca243, F124, F37, Fca075, Fca559, Fca057, Fca081, Fca566, F42, Fca043, Fca161, Fca293, Fca369, and Fca668) identified by Menotti-Raymond et al.^59,60^. Methods for the amplification, sequencing, data quality testing, and genotyping are provided in *SI *Appendix 1.

We assessed genotype data conformance to assumptions of Hardy–Weinberg equilibrium [HWE], linkage disequilibrium [LD], and null alleles with a subset of 161 adult and subadult radiocollared panthers to avoid over-representation of alleles from previously sampled kittens handled at maternal dens that were related to this subsample (*SI *Appendix 1). Tests for HWE and LD were completed in GENEPOP version 4.7^61^; tests for null alleles were completed in MICROCHECKER 2.2.3^62^. Similar analyses were completed on samples collected from more contiguous puma populations in Colorado, Idaho, North Dakota, South Dakota, and Texas (Western puma) for comparison (*SI *Appendix 1)*.*

### Assessment of genetic ancestry and delineations of panther cohorts

We assessed the effect of genetic ancestry and year-of-birth cohorts (hereafter, cohorts) on variables that are associated with genetic variation and genetic structure within panthers and among populations of Western puma. We used genotype data from the 16 microsatellite loci to implement a Bayesian clustering analysis in Program STRUCTURE version 2.3.4^30^ to infer ancestral clusters of sampled adult and subadult panthers (see *SI *Appendix 2* for detailed* STRUCTURE *analysis methods and for* STRUCTURE results from the complete data set that includes kittens). We then used *q*-values provided in runs for the best *K* (number of genetic clusters; for this analysis *K* = 2) to assign individual panthers as either canonical (≥ 90% pre-introgression ancestry) or admixed (< 90% pre-introgression ancestry) ancestry. For some analyses, we focused interpretations on subadult and adult panthers we sampled and did not include kittens handled only at maternal dens. Given the high annual mortality estimated for kittens 0.323^17^, it makes sense to focus population-wide changes in ancestry composition on the age groups that are more likely to contribute to subsequent generations of panthers.

To assess the temporal persistence of genetic benefits across generations, we delineated five cohorts of panthers known or estimated to have been born within a certain timeframe: pre-genetic rescue group 1 (PRE1) inclusive of panthers born < 1986; PRE2, 1986–1995; post-genetic rescue 1 (POST1), 1996–2005; POST2, 2006–2015, and POST3, 2016–2020. This permitted us to categorize panthers in groups that did not rely on ancestral designation from STRUCTURE but instead provided insight into the changes in metrics of genetic health of the panther population through time.

### Morphological and biomedical correlates of inbreeding

During each panther capture or necropsy we recorded the presence or absence of correlates of inbreeding depression^9,12^ that included kinked tails resulting from a deformity of the distal caudal vertebrae; cowlicks that are a reversal of hair direction on the midline dorsum, cryptorchidism, ASDs, and percent structurally abnormal spermatozoa sperm data from Penfold et al.^46^. Statistical comparisons among the proportions or mean percentage of these traits in each ancestral category or cohort were completed in Program R version 4.2.1^63^; tests are described in detail in *SI *Appendix 3.

### Measures of genetic variation

We analyzed genotypes from all 16 loci for a subset of adult and subadult panthers (N = 552) for analyses of genetic variation and population genetic structure within panthers and between populations of Western puma. Genotypes of panther kittens that were only sampled at maternal dens were not used in these analyses because related individuals are known to result in the over-representation of certain alleles^64^. We used GenAlex 6.5^33,34^ to determine the number of alleles (N_a_), number of effective alleles (N_effective_), observed heterozygosity (H_o_), and expected heterozygosity (H_e_). We used hp-rare^65^ to estimate allelic richness (A_r_) using the rarefaction technique to account for different sample sizes among groups of panthers and Western pumas. Statistical analyses to assess among-group differences for these metrics are detailed in *SI *Appendix 4.

Inbreeding depression in wild populations is commonly assessed using heterozygosity–fitness correlations (HFC), although their application in endangered populations is less common^52^. We estimated the heterozygosity of each individual panther by first calculating homozygosity by loci (HL), which varies between 0 (all loci heterozygous) and 1 (all loci homozygous)^66^ using the Rhh package^67^ in Program R. We then calculated individual heterozygosity (H_ind_) for each panther as 1–HL (see^45^), thereby creating a scale where values increase as an individual approaches having a genotype that is completely heterozygous^68^. We used linear regression to quantify among-group differences in mean H_ind_ separately for (a) panther ancestral groups and Western pumas, and (b) panther cohorts and Western pumas. For the HFC analysis, we used logistic regression to quantify among-group (ancestral, cohorts and Western pumas) differences in the probability of expressing a correlate of inbreeding and to estimate the influence of H_ind_, a continuous predictor variable, on the probability of trait expression. Additional details on statistical analyses are provided in *SI *Appendix 5*.*

### Genetically effective population size, abundance, and principal coordinate analysis

We calculated N_e_ by applying the LD method^31,69, 70^ within NeEstimator 2.1^32^ using the random mating model and did not accept singleton alleles, since these are known to contribute to an upward bias of N_e_ estimates^32^. This method also provided 95% confidence intervals with estimates of N_e_ using both parametric and a jackknife over individuals methods^71^.

Estimating the abundance of large carnivores is difficult and has been a challenge for panther managers. Agencies involved in panther conservation have relied on minimum counts of panthers accrued via indirect field evidence (inventory of sign) and directly from captures^10^, but this method required extensive effort and the counts did not: (1) provide a population estimate, (2) incorporate detection coefficients, and (3) have an associated measure of error. To ameliorate those issues and provide a more statistically robust estimate, we applied the methodology of McClintock et al.^72^, with some minor adjustments (see *SI *Appendix 6), to estimate the population size during the post-genetic restoration period of 2000–2018. We used these estimates, the minimum population counts of McBride et al.^10^ for the period 1981–2015^19^, and the population estimate derived from an integrated population model from Merriell^73^ for the period 1982–2020 to assess trends in the population size pre- and post-genetic rescue.

We conducted PCoA plots to visually represent the differences between allele frequencies at different sampling locations, cohorts, and ancestry and to assess the level of genetic distinctness of panthers compared to Western puma populations. PCoA is a multivariate technique that permits the plotting of patterns in a multivariate data set (e.g., multiple microsatellite loci for multiple samples). We completed two separate PCoA using GenAlEx 6.5^33,34^ with microsatellite genotypes of panthers categorized by ancestry and by cohort (Pre1, Pre2, Post 1, Post 2, Post 3) and plotted those with genotype data from Western pumas. A parallel analysis using the more traditional fixation index (F_st_) to assess pairwise genetic structure among groups of panthers and Western pumas was also completed in GenAlex 6.5^33,34^.

### Reporting summary

Further information on research design is available in the Nature Portfolio Reporting Summary linked to this article.

### Supplementary Information

Supplementary Information 1.

Supplementary Legends.

Supplementary Figure 1.

Supplementary Figure 2.

Supplementary Figure 3.

## Data Availability

All study data are included in the article and/or Supplemental Information.

## References

[1] Di Minin, E. *et al.* Global priorities for national carnivore conservation under land use change. *Sci. Rep.* **6**, 23814 (2016). 27034197 10.1038/srep23814 PMC4817124

[2] Ripple, W. J. *et al.* Status and ecological effects of the world’s largest carnivores. *Science* **343**, 1241484 (2014). 24408439 10.1126/science.1241484

[3] Gilpin, M. & Soulé, M. E. *Conservation Biology: The Science of Scarcity and Diversity* 19–34 (Sinauer Associates, 1986).

[4] Whiteley, A. R., Fitzpatrick, S. W., Funk, W. C. & Tallmon, D. A. Genetic rescue to the rescue. *Trends Ecol. Evol.* **30**, 42–49 (2015). 25435267 10.1016/j.tree.2014.10.009

[5] Vila, C. *et al.* Rescue of a severely bottlenecked wolf (*Canis lupus*) population by a single immigrant. *Proc. R. Soc. Lond. B* **270**, 91–97 (2003). 10.1098/rspb.2002.2184 PMC1691214 12590776

[6] Bouzat, J. L. *et al.* Beyond the beneficial effects of translocations as an effective tool for the genetic restoration of isolated populations. *Conserv. Genet.* **10**, 191–201 (2009). 10.1007/s10592-008-9547-8

[7] Capel, S. L. R. *et al.* Evaluating the genome-wide impacts of species translocations: The greater prairie-chicken as a case study. *Conserv. Genet.* **23**, 179–191 (2022). 10.1007/s10592-021-01412-8

[8] Smyser, T. J., Johnson, S. A., Page, L. K., Hudson, C. M. & Rhodes, O. E. Use of experimental translocations of Allegheny woodrat to decipher causal agents of decline. *Conserv. Biol.* **27**, 752–762 (2013). 23647164 10.1111/cobi.12064

[9] Onorato, D. *et al.**Biology and Conservation of Wild Felids* 453–469 (Oxford University Press, 2010).

[10] McBride, R. T., McBride, R. T., McBride, R. M. & McBride, C. E. Counting pumas by categorizing physical evidence. *Southeast. Nat.* **7**, 381–400 (2008). 10.1656/1528-7092-7.3.381

[11] Culver, M., Hedrick, P. W., Murphy, K., O’Brien, S. & Hornocker, M. G. Estimation of the bottleneck size in Florida panthers. *Anim. Conserv.* **11**, 104–110 (2008). 10.1111/j.1469-1795.2007.00154.x

[12] Roelke, M. E., Martenson, J. S. & O’Brien, S. J. The consequences of demographic reduction and genetic depletion in the endangered Florida panther. *Curr. Biol.* **3**, 340–349 (1993). 15335727 10.1016/0960-9822(93)90197-V

[13] Barone, M. A. *et al.* Reproductive characteristics of male Florida panthers: Comparative studies from Florida, Texas, Colorado, Latin-America, and North-American Zoos. *J. Mammal.* **75**, 150–162 (1994). 10.2307/1382247

[14] Seal, U. S. *A Plan for Genetic Restoration and Management of the Florida Panther (Felis concolor coryi)* (Apple Valley, 1994).

[15] Benson, J. F. *et al.* Intentional genetic introgression influences survival of adults and subadults in a small, inbred felid population. *J. Anim. Ecol.* **80**, 958–967 (2011). 21338353 10.1111/j.1365-2656.2011.01809.x PMC6993175

[16] Hostetler, J. A., Onorato, D. P., Jansen, D. & Oli, M. K. A cat’s tale: The impact of genetic restoration on Florida panther population dynamics and persistence. *J. Anim. Ecol.* **82**, 608–620 (2013). 23252671 10.1111/1365-2656.12033

[17] Hostetler, J. A. *et al.* Genetic introgression and the survival of Florida panther kittens. *Biol. Conserv.* **143**, 2789–2796 (2010). 21113436 10.1016/j.biocon.2010.07.028 PMC2989677

[18] Johnson, W. E. *et al.* Genetic restoration of the Florida panther. *Science* **329**, 1641–1645 (2010). 20929847 10.1126/science.1192891 PMC6993177

[19] McBride, R. & McBride, C. *Florida Panther Annual Count 2015* (Rancher’s Supply Inc., 2015).

[20] Tallmon, D. A., Luikart, G. & Waples, R. S. The alluring simplicity and complex reality of genetic rescue. *Trends Ecol. Evol.* **19**, 489–496 (2004). 16701312 10.1016/j.tree.2004.07.003

[21] Bell, D. A. *et al.* The exciting potential and remaining uncertainties of genetic rescue. *Trends Ecol. Evol.* **34**, 1070–1079 (2019). 31296345 10.1016/j.tree.2019.06.006

[22] Willi, Y. *et al.* Conservation genetics as a management tool: The five best-supported paradigms to assist the management of threatened species. *Proc. Natl. Acad. Sci.* **119**, e2105076119 (2022). 34930821 10.1073/pnas.2105076119 PMC8740573

[23] Pérez-Pereira, N., Caballero, A. & García-Dorado, A. Reviewing the consequences of genetic purging on the success of rescue programs. *Conserv. Genet.* 23, 1–17 (2022). 10.1007/s10592-021-01405-7

[24] Hwang, A. S., Northrup, S. L., Alexander, J. K., Vo, K. T. & Edmands, S. Long-term experimental hybrid swarms between moderately incompatible *Tigriopus californicus* populations: Hybrid inferiority in early generations yields to hybrid superiority in later generations. *Conserv. Genet.* **12**, 895–909 (2011). 10.1007/s10592-011-0193-1

[25] Hufbauer, R. A. *et al.* Three types of rescue can avert extinction in a changing environment. *Proc. Natl. Acad. Sci.* **112**, 10557–10562 (2015). 26240320 10.1073/pnas.1504732112 PMC4547288

[26] Crow, J. F. Alternative hypotheses of hybrid vigor. *Genetics* **33**, 477 (1948). 17247292 10.1093/genetics/33.5.477 PMC1209419

[27] Frankham, R. Genetic rescue of small inbred populations: Meta-analysis reveals large and consistent benefits of gene flow. *Mol. Ecol.* **24**, 2610–2618 (2015). 25740414 10.1111/mec.13139

[28] Frankham, R. Genetic rescue benefits persist to at least the F3 generation, based on a meta-analysis. *Biol. Conserv.* **195**, 33–36 (2016). 10.1016/j.biocon.2015.12.038

[29] Charlesworth, B. Effective population size and patterns of molecular evolution and variation. *Nat. Rev. Genet.* **10**, 195–205 (2009). 19204717 10.1038/nrg2526

[30] Pritchard, J. K., Stephens, M. & Donnelly, P. Inference of population structure using multilocus genotype data. *Genetics* **155**, 945–959 (2000). 10835412 10.1093/genetics/155.2.945 PMC1461096

[31] Waples, R. S. & Do, C. Linkage disequilibrium estimates of contemporary Ne using highly variable genetic markers: A largely untapped resource for applied conservation and evolution. *Evol. Appl.* **3**, 244–262 (2010). 25567922 10.1111/j.1752-4571.2009.00104.x PMC3352464

[32] Do, C. *et al.* NeEstimator v2: Re-implementation of software for the estimation of contemporary effective population size (Ne) from genetic data. *Mol. Ecol. Resourc.* **14**, 209–214 (2014). 10.1111/1755-0998.12157 23992227

[33] Peakall, R. & Smouse, P. E. GENALEX 6: Genetic analysis in Excel. Population genetic software for teaching and research. *Mol. Ecol. Resourc.* **6**, 288–295 (2006). 10.1111/j.1471-8286.2005.01155.x PMC3463245 22820204

[34] Peakall, R. & Smouse, P. E. GenAlEx 6.5: Genetic analysis in Excel. Population genetic software for teaching and research: An update. *Bioinformatics* **28**, 2537–2539 (2012). 22820204 10.1093/bioinformatics/bts460 PMC3463245

[35] Hedrick, P. W. & Garcia-Dorado, A. Understanding inbreeding depression, purging, and genetic rescue. *Trends Ecol. Evol.* **31**, 940–952 (2016). 27743611 10.1016/j.tree.2016.09.005

[36] Maehr, D. S. & Caddick, G. B. Demographics and genetic introgression in the Florida panther. *Conserv. Biol.* **9**, 1295–1298 (1995). 34261271 10.1046/j.1523-1739.1995.9051288.x-i1

[37] Harris, K., Zhang, Y. & Nielsen, R. Genetic rescue and the maintenance of native ancestry. *Conserv. Genet.* **20**, 59–64 (2019). 10.1007/s10592-018-1132-1

[38] Fitzpatrick, S. W. *et al.* Genomic and fitness consequences of genetic rescue in wild populations. *Curr. Biol.* **30**, 517–522 (2020). 31902732 10.1016/j.cub.2019.11.062

[39] Frankham, R., Ballou, J. D. & Briscoe, D. A. *Introduction to Conservation Genetics* (Cambridge University Press, 2002).

[40] Wilkins, L., Arias-Reveron, J. M., Stith, B., Roelke, M. E. & Belden, R. C. The Florida panther: A morphological investigation of the subspecies with a comparison to other North and South American cougars. *Bull. Fla. Mus. Nat. Hist.* **40**, 221–269 (1997).

[41] Cunningham, M. W. *et al.* Atrial septal defects in Florida panthers. *J. Wildl. Dis.* **35**, 519–530 (1999). 10479086 10.7589/0090-3558-35.3.519

[42] Räikkönen, J., Vucetich, J. A., Vucetich, L. M., Peterson, R. O. & Nelson, M. P. What the inbred Scandinavian wolf population tells us about the nature of conservation. *PLoS ONE* **8**, e67218 (2013). 23805301 10.1371/journal.pone.0067218 PMC3689695

[43] Lioy, F. G. *et al.* Show me your tail, if you have one! Is inbreeding depression occurring in wildcats (*Felis silvestris silvestris*) from Italy?. *Mamm. Res.* **67**, 153–161 (2022). 10.1007/s13364-022-00627-5

[44] DeRose, M. A. & Roff, D. A. A comparison of inbreeding depression in life-history and morphological traits in animals. *Evolution* **53**, 1288–1292 (1999). 28565531 10.2307/2640831

[45] van de Kerk, M., Onorato, D. P., Hostetler, J. A., Bolker, B. M. & Oli, M. K. Dynamics, persistence, and genetic management of the endangered Florida panther population. *Wildl. Monogr.* **203**, 3–35 (2019). 10.1002/wmon.1041

[46] Penfold, L. M. *et al.* Long-term evaluation of male Florida panther (*Puma concolor coryi*) reproductive parameters following genetic introgression. *J. Mammal.* **103**, 835–844 (2022). 10.1093/jmammal/gyac029

[47] Gustafson, K. D. *et al.* Genetic source–sink dynamics among naturally structured and anthropogenically fragmented puma populations. *Conserv. Genet.* **20**, 215–227 (2019). 10.1007/s10592-018-1125-0

[48] Holbrook, J. D. *et al.* Genetic diversity, population structure, and movements of mountain lions (*Puma concolor*) in Texas. *J. Mammal.* **93**, 989–1000 (2012). 10.1644/11-MAMM-A-326.2

[49] Gustafson, K. D. *et al.* Multi-population puma connectivity could restore genomic diversity to at-risk coastal populations in California. *Evol. Appl.* **15**, 286–299 (2022). 35233248 10.1111/eva.13341 PMC8867711

[50] Ruiz-López, M. J. *et al.* Heterozygosity-fitness correlations and inbreeding depression in two critically endangered mammals. *Conserv. Biol.* **26**, 1121–1129 (2012). 22897325 10.1111/j.1523-1739.2012.01916.x

[51] Johnson, H. E., Mills, L. S., Wehausen, J. D., Stephenson, T. R. & Luikart, G. Translating effects of inbreeding depression on component vital rates to overall population growth in endangered bighorn sheep. *Conserv. Biol.* **25**, 1240–1249 (2011). 22070275 10.1111/j.1523-1739.2011.01739.x

[52] Grueber, C. E., Wallis, G. P. & Jamieson, I. G. Heterozygosity–fitness correlations and their relevance to studies on inbreeding depression in threatened species. *Mol. Ecol.* **17**, 3978–3984 (2008). 19238701 10.1111/j.1365-294X.2008.03910.x

[53] Lamb, C. T. *et al.* The ecology of human-carnivore coexistence. *Proc. Natl. Acad. Sci.* **117**, 17876–17883 (2020). 32632004 10.1073/pnas.1922097117 PMC7395549

[54] Ordiz, A., Bischof, R. & Swenson, J. E. Saving large carnivores, but losing the apex predator?. *Biol. Conserv.* **168**, 128–133 (2013). 10.1016/j.biocon.2013.09.024

[55] Ralls, K. *et al.* Call for a paradigm shift in the genetic management of fragmented populations. *Conserv. Lett.* **11**, e12412 (2018). 10.1111/conl.12412

[56] Robinson, Z. L. *et al.* Evaluating the outcomes of genetic rescue attempts. *Conserv. Biol.* **35**, 666–677 (2021). 32700770 10.1111/cobi.13596

[57] FWC. *Annual Report on the Research and Management of Florida Panthers: 2020–2021*. (Fish and Wildlife Research Institute & Division of Habitat and Species Conservation; Florida Fish and Wildlife Conservation Commission, 2021).

[58] Sikes, R. S. 2016 Guidelines of the American Society of Mammalogists for the use of wild mammals in research and education. *J. Mammal.* **97**, 663–688 (2016). 29692469 10.1093/jmammal/gyw078 PMC5909806

[59] Menotti-Raymond, M. *et al.* A genetic linkage map of microsatellites in the domestic cat (*Felis catus*). *Genomics* **57**, 9–23 (1999). 10191079 10.1006/geno.1999.5743

[60] Menotti-Raymond, M. *et al.* An autosomal genetic linkage map of the domestic cat, *Felis silvestris catus*. *Genomics* **93**, 305–313 (2009). 19059333 10.1016/j.ygeno.2008.11.004 PMC2656606

[61] Rousset, F. Genepop’007: A complete re-implementation of the genepop software for Windows and Linux. *Mol. Ecol. Resourc.* **8**, 103–106 (2008). 10.1111/j.1471-8286.2007.01931.x 21585727

[62] Van Oosterhout, C., Hutchinson, W. F., Wills, D. P. & Shipley, P. MICRO-CHECKER: Software for identifying and correcting genotyping errors in microsatellite data. *Mol. Ecol. Resourc.* **4**, 535–538 (2004). 10.1111/j.1471-8286.2004.00684.x

[63] *R: A Language and Environment for Statistical Computing* (R Foundation for Statistical Computing, 2022).

[64] Marshall, T. C., Slate, J., Kruuk, L. E. B. & Pemberton, J. M. Statistical confidence for likelihood-based paternity inference in natural populations. *Mol. Ecol.* **7**, 639–655 (1998). 9633105 10.1046/j.1365-294x.1998.00374.x

[65] Kalinowski, S. T. HP-RARE 1.0: A computer program for performing rarefaction on measures of allelic richness. *Mol. Ecol. Notes* **5**, 187–189 (2005). 10.1111/j.1471-8286.2004.00845.x

[66] Aparicio, J. M., Ortega, J. & Cordera, P. J. What should we weigh to estimate heterozygosity, alleles or loci?. *Mol. Ecol.* **15**, 4659–4665 (2006). 17107491 10.1111/j.1365-294X.2006.03111.x

[67] Alho, J. S., Välimäki, K. & Merilä, J. Rhh: An R extension for estimating multilocus heterozygosity and heterozygosity-heterozygosity correlation. *Mol. Ecol. Resourc.* **10**, 720–722 (2010). 10.1111/j.1755-0998.2010.02830.x 21565077

[68] Ferrer, E. S., García-Navas, V., Sanz, J. J. & Ortego, J. The strength of the association between heterozygosity and probability of interannual local recruitment increases with environmental harshness in blue tits. *Ecol. Evol.* **6**, 8857–8869 (2016). 28035274 10.1002/ece3.2591 PMC5192745

[69] Hill, W. G. Estimation of effective population size from data on linkage disequilibrium. *Genet. Res.* **38**, 209–216 (1981). 10.1017/S0016672300020553

[70] Waples, R. S. A bias correction for estimates of effective population size based on linkage disequilibrium at unlinked gene loci. *Conserv. Genet.* **7**, 167–184 (2006). 10.1007/s10592-005-9100-y

[71] Jones, A. T., Ovenden, J. R. & Wang, Y. G. Improved confidence intervals for the linkage disequilibrium method for estimating effective population size. *Heredity* **117**, 217–223 (2016). 27005004 10.1038/hdy.2016.19 PMC5026751

[72] McClintock, B. T., Onorato, D. P. & Martin, J. Endangered Florida panther population size determined from public reports of motor vehicle collision mortalities. *J. Appl. Ecol.* **52**, 893–901 (2015). 10.1111/1365-2664.12438

[73] Merriell, B. D. *Demography and Population Dynamics of the Florida Panther: An Integrated Population Modeling Approach* (University of Florida, 2021).

[74] Juarez, R. L. *et al.* Assessing temporal genetic variation in a cougar population: Influence of harvest and neighboring populations. *Conserv. Genet.* **17**, 379–388 (2016). 10.1007/s10592-015-0790-5

