# Supplementary material for: Multi-generational benefits of genetic rescue
Source: Sci Rep. 2024 Jul 30;14:17519. doi: 10.1038/s41598-024-67033-6 (PMC11289468; doi:10.1038/s41598-024-67033-6)
Supplement: Supplementary file 1 — Supplementary Information 1. [file 41598_2024_67033_MOESM1_ESM.pdf]

Scientific Reports

Supplemental Information for-

Multi-generational benefits of genetic rescue

Dave P. Onorato[a1], Mark W. Cunningham[b], Mark Lotz[c], Marc Criffield[a], David Shindle[d], Annette Johnson[e], Bambi C. F. Clemons[b], Colin P. Shea[f], Melody E. Roelke-Parker[g], Warren E. Johnson[h], Brett T. McClintock[i], Kristine L. Pilgrim[j], Michael K. Schwartz[j], Madan K. Oli[k]

Corresponding Author: Dave Onorato dave.onorato@myfwc.com

Supplemental Information Appendix 1- Laboratory protocols and microsatellite assessment

Methods

All laboratory analyses on panther DNA samples were completed at the National Genomics Center for Wildlife and Fish Conservation (NGCWFC, Missoula, MT, USA). We amplified DNA extractions in 10-µl reaction volumes that included 1.0 µl of DNA, 1X reaction buffer (Life Technologies Corporation, Grand Island, NY, USA), 2.0 mM of $MgCl_2$, 200 µM of each dNTP, 1 µM of reverse primer, 1 µM of dye-labeled forward primer, 1.5 mg/ml of bovine serum albumin (BSA), and 1U Taq polymerase (Life Technologies). The polymerase chain reactions (PCRs) included a thermal profile of 94°C for 5 minutes followed by 36 cycles of 94°C for 60 seconds, 55°C for 60 seconds, and 72°C for 30 seconds. Resulting PCR products were visualized on an LI-COR DNA analyzer (LI-COR Biotechnology, Lincoln, NE, USA). We collected genotypes from Florida panther hair (n = 19) and saliva (n= 8) samples using a multitube approach [1], error-checked genotypes using Program DROPOUT [2], and combined them with genotypes from panther tissue and blood samples (n = 877).

We used GENEPOP version 4.7 to test for: 1) deviations from HWE via F_{IS} [3] using the exact test methodology Guo and Thompson [4]; and 2) pairwise linkage disequilibrium [5,6]. These analyses were completed on a subset of 161 adult and subadult radiocollared panthers to reduce the overrepresentation of alleles from kittens and other known adult/subadult relatives in the larger sample. We used the unbiased estimate of Fisher's exact test via the Markov chain method with the settings of 1,000 dememorization, 1,000 batches, and 10,000 iterations. We considered deviations from HWE (0.05/16 = 0.003125) and LD (0.05/([16*15]/2 = 0.0004) as significant when critical values were adjusted for the likelihood of making a Type-I error using the sequential Bonferroni *P*-value corresponding to alpha = 0.05 [7]. We tested for the presence of null alleles using the program MICROCHECKER 2.2.3 [8] using the null allele frequencies calculated via the method of Chakraborty et al. [9]. For comparative purposes in some analyses, we used genotype data from the same 16 loci for pumas from Texas (n = 47), the source population for the genetic rescue program, as well as pumas from Colorado (n = 23), Idaho (n = 23), North Dakota (n = 22), and South Dakota (n = 26). These data were provided to us by the NGCWFC.

Results

Results for some of these analyses are presented in Table A1.1-A1.2. For panthers, 6 of the 16 loci exhibited F_{IS} values that indicated they deviated significantly from HWE (FCA090, FCA243, F124, FCA057, F42, FCA369) after adjusting the *P*-value with the Bonferroni correction (P_{crit} = 0.05/16 loci or 0.0031; Table A1.1). Five of these loci had positive F_{IS} values that denote heterozygote deficiency. By comparison, only 1 locus significantly deviated from HWE expectations for Colorado (F124), North

Dakota (FCA133), and Texas (FCA369); there were no loci out of HWE in the South Dakota and Idaho populations. For panthers, there was significant evidence of LD in 45 out of 120 pairwise comparisons between 16 loci after adjusting the critical *P*-value with the Bonferroni correction (P_{crit} = 0.05/120 pairwise comparisons or 0.000417). Only 8 of the 45 pairwise comparisons involved loci that were on the same chromosomes, suggesting that physical linkage was not an issue in 82% of the pairwise comparisons where LD was significant. None of the Western populations of pumas had any pairwise comparisons between loci that exhibited significant evidence of LD except in one case in the North Dakota population (1 of 120 pairwise comparisons). The potential for the presence of null alleles was identified at 5 (FCA090, FCA243, F124, FCA559, FCA668) of 16 loci in panthers (Table A1.2). Comparatively, only 1-3 loci had the potential for the presence of null alleles in each of the Western populations of puma (Table A1.2)

There are a variety of reasons we observed deviations from HWE, heterozygote deficiencies, LD, and null alleles in our microsatellite analyses on Florida panther samples. Most can be attributed to the population bottleneck and associated low levels of genetic variation in the population noted in the 1980s and early 1990s [10,11]. Deviations from HWE expectations would not be unexpected when multiple assumptions of HWE are violated. Inbreeding, non-random mating, and population fragmentation are all causes of nonconformities from HWE and are factors that certainly impacted the Florida panther in the past and present. Inbreeding was documented in previous genetic analyses [10,11], along with a population bottleneck [12]. Additionally, the panther population remains isolated (i.e., fragmented) from other breeding populations of puma to the West. Similarly, the level of LD in our sample of Florida panthers can be attributed to many of the same factors. Linkage disequilibrium is expected to be common in small, isolated populations and those that have been impacted by a bottleneck [13]. Three loci that were noted as potentially exhibiting null alleles (FCA090, FCA243, and F124) also were identified as being impacted by heterozygote deficiency in HWE tests (positive F_{IS} values). Inbreeding, as previously noted, can result in a depletion of genetic variation in a population over time and lead to a preponderance of homozygotes at certain loci. Shirk et al. [14] noted this issue in their null allele analyses of mountain goat (*Oreamnos americanus*) microsatellite data and retained several loci that indicated the potential presence of null alleles due to the impact of inbreeding on their subpopulations.

Nevertheless, non-conformance to these parameters at some loci is typically an indicator that genotypes from those markers may need to be used with caution or not applied in subsequent analyses. To determine if the deviations we observed were directly related to the loci that we selected or an artifact of the historical population genetics of Florida panthers, we completed similar tests on genotypes from the same 16 loci that were amplified from 141 puma collected from 5 larger Western populations. Those analyses revealed fewer deviations from HWE (6 of 16 loci for panthers vs. 3 total for the other 5 populations), one instance of LD out of 600 pairwise comparisons, and a reduction in the potential presence of null alleles (Table A1.1-A1.2). These results provide support for our hypothesis that deviations of certain metrics in our genotype data from Florida panthers is most likely associated with the demographic history of this isolated population prior to genetic rescue and not a result of problematic loci, justifying our application of these data for our analyses of these and the Western puma samples.

Table A1.1. Descriptive statistics regarding the conformance of genotypes collected from Florida panther tissue samples at 16 microsatellite loci to assumptions associated with Hardy-Weinberg equilibrium. Texas pumas and other populations of pumas are presented for comparative purposes to demonstrate that non-conformance of some loci in Florida panthers is not associated with the loci themselves but instead is related to the genetic history of the panther population.

	Florida panther (n=161)		Texas puma (n=47)		Colorado puma (n=23)		Idaho puma (n=23)		North Dakota puma (n=22)		South Dakota puma (n=26)	
Locus	F_{is}	*P*-value[a]	F_{is}	*P*-value[a]	F_{is}	*P*-value[a]	F_{is}	*P*-value[a]	F_{is}	*P*-value[a]	F_{is}	*P*-value[a]
FCA090	0.1862	**0.002***	-0.001	0.0954	0.0421	0.1575	0.1254	0.1409	0.0711	0.8705	-0.0239	0.3031
FCA133	0.0515	0.0939	-0.2224	0.1179	-0.1687	0.8241	-0.0203	0.6205	0.6358	**0.0018***	-0.2048	0.1993
FCA243	0.1997	**0.0003***	0.0722	0.6873	0.1504	0.6434	-0.0108	0.619	0.2617	0.0924	0.2768	0.0065
F124	0.121	**0.0011***	0.1954	0.0796	0.5713	**0.0000***	0.3247	0.0144	0.003	0.3198	0.3545	0.0142
F37	0.0247	0.1593	0.1126	0.0433	-0.1873	0.8215	0.2474	0.2053	-0.2069	0.6285	0.0741	0.1601
FCA075	0.0785	0.0843	-0.0675	0.0988	-0.0798	0.2686	-0.2343	0.2562	0.0294	1.0000	0.0741	0.0047
FCA559	0.1144	0.043	-0.0731	0.1022	0.1055	0.73	-0.3091	0.201	0.1318	0.6868	-0.0225	0.7607
FCA057	-0.0098	**0.002***	-0.0781	0.6682	0.2048	0.2344	0.3141	0.0039	-0.0311	0.4999	0.1607	0.0567
FCA081	-0.0289	0.829	0.0544	0.8998	0.3393	0.0139	-0.0936	0.1023	0.1829	0.0361	0.0037	0.3049
FCA566	0.0657	0.1117	0.0208	0.7165	-0.3968	0.1666	-0.4408	0.039	-0.3049	0.7024	0.1405	0.0926
F42	0.0419	**0.0000***	0.0141	0.1127	0.0685	0.1576	-0.0409	0.9528	0.0442	0.5503	0.0724	0.3933
FCA043	-0.0237	0.8644	-0.0153	0.4423	-0.0423	0.4661	-0.2571	0.5484	-0.01	1.0000	0.0669	0.1047
FCA161	0.0359	0.0838	0.0471	0.7875	-0.1821	0.7235	0.0585	1.0000	-0.0328	0.2148	-0.0475	0.8375
FCA293	0.0488	0.0248	0.045	0.6511	0.1837	0.1379	0.2657	0.0559	0.4362	0.0192	0.3785	0.0083
FCA369	0.0554	**0.0003***	0.0391	**0.0014***	-0.2263	0.8408	-0.0804	1.0000	-0.0787	0.5415	-0.0274	1.0000
FCA668	0.1418	0.0056	-0.0161	0.8498	0.2281	0.0055	0.2667	0.2075	-0.1053	1.0000	-0.1421	0.8307

[a] Critical *P*-value when adjusted via the Bonferroni correction is 0.05/16 (loci) or $P < 0.0031$.
[*] Loci that deviate significantly from HWE after Bonferroni correction.

Table A1.2 Presence of null alleles at 16 microsatellite loci used on Florida panthers. Texas pumas and other populations of pumas are presented for comparative purposes to demonstrate that the presence of null alleles at loci in Florida panthers is not associated with the loci themselves but instead is related to the genetic history of the panther population.

Locus	Florida panther (n=161)	Texas puma (n=47)	Colorado puma (n=23)	Idaho puma (n=23)	North Dakota puma (n=22)	South Dakota puma (n=26)
FCA090	**0.1008***	-0.0059	0.01	0.0545	0.0245	-0.0213
FCA133	0.0248	-0.1042	-0.0873	-0.0208	**0.4511***	-0.1005
FCA243	**0.1091***	0.0315	0.0675	-0.0162	0.1363	**0.1485***
F124	**0.0626***	**0.102***	**0.3851***	**0.1797***	-0.01	**0.2028***
F37	0.0109	0.0537	-0.0945	0.1276	-0.1028	0.028
FCA075	0.0391	-0.0377	-0.0484	-0.1132	0.0031	0.028
FCA559	**0.0589***	-0.0402	0.0435	-0.1413	0.0576	-0.0206
FCA057	-0.0064	-0.0426	0.101	**0.1723***	-0.0264	0.0761
FCA081	-0.0157	0.0221	**0.1901***	-0.0546	0.0871	-0.0079
FCA566	0.0323	0.0048	-0.172	-0.1868	-0.1401	0.0646
F42	0.0198	0.0016	0.0237	-0.031	0.0106	0.0271
FCA043	-0.0133	-0.0128	-0.0312	-0.122	-0.0164	0.0242
FCA161	0.0167	0.0183	-0.0923	0.0185	-0.0272	-0.0324
FCA293	0.0233	0.0173	0.087	**0.1396***	**0.2636***	**0.2206***
FCA369	0.0268	0.0143	-0.1104	-0.0487	-0.0484	-0.023
FCA668	**0.0745***	-0.0134	0.1154	0.1402	-0.0602	-0.0746

*Locus that shows evidence for a null allele. Null allele frequency calculated using methods of Chakraborty et al. [9].

Supplemental Information Appendix 2- STRUCTURE analysis of ancestry

Methods

We used genotype data from the 16 microsatellite loci to implement a Bayesian clustering analysis in Program STRUCTURE version 2.3.4 [15] to infer ancestral clusters of 904 sampled adult, subadult, and dependent-aged Florida panthers, 49 Texas pumas (inclusive of 7 of the 8 females introduced in 1995), and 12 other non-Florida pumas. This method uses a Markov chain Monte Carlo (MCMC) method to determine the number of genetic clusters (K) in the sample while also providing an individual's percentage of ancestry (q-values) allocated to each cluster [15]. Ten iterations of these data at each K = 1-10 runs with a 100,000 MCMC burn-in period followed by 500,000 MCMC iterations. To determine the most likely number of K genetic clusters, we used the logarithm of the probability of the data lnP(D)|K; ref. [15] and estimates of ΔK [16] in program STRUCTURE HARVESTER [17]. We then used q-values provided in runs for the best K (which was 2 in this analysis) to assign individual panthers as either canonical (≥90% pre-introgression ancestry) or admixed (<90% pre-introgression ancestry) ancestry.

Results

Visualization of two cluster model of genetic population structure in adult and subadult Florida panthers

For improved visualization of our results with regards to the admixture associated with genetic rescue, we first present the results of our STRUCTURE clustering analysis of ancestry that focuses on sub-adult and adult panthers we handled (n = 547) and excludes kittens documented at natal dens. Given the low annual survival rate estimated for Florida panther kittens 0.323; [18], it makes sense to focus population wide changes in ancestry composition to the age groups that are more likely to contribute to subsequent generations of panthers. Our analysis supported a two-cluster model (ΔK = 1786 at K = 2, Ln likelihood $_{(K=2)}$ = -31951, sd = 0.8) from which we used q-values (proportion of an individual's genome [i.e., ancestry] originating from a cluster in K) to assign panthers as either canonical (≥90% pre-introgression ancestry) or admixed (<90% pre-introgression ancestry). See Table A2.1. below. The homogeneous nature of ancestry in panthers is evident in **Extended Data Fig. 1 Panels A and B** (Pre1 and Pre2 cohorts of panthers), a period when the population was at its nadir and comprised of highly inbred canonical panthers. Subsequently, after the release of 8 female pumas from Texas in 1995 (note the 7 Texas females we sampled are indicated as stippled red bars at the beginning of panel C in both figures), we see significant increases in the levels of admixture in cohorts of panthers born in the Post1-3 periods (panels C-E).

Table A2.1. Ancestry of adult and subadult Florida panthers (n = 547) sampled 1981–2020 in Florida, USA. Ancestry was determined via a clustering analysis of microsatellite genotype data using program STRUCTURE with $K = 2$ clusters (canonical and admixed). Cohorts include Florida panthers born during the pre-genetic rescue (Pre1 and Pre2) and post-genetic rescue (Post1-3) periods. Panthers were designated as canonical if their canonical ancestry *q*-value was ≥ 90%. Mean *q*-values for each ancestry category are presented to demonstrate changes in the composition of population ancestry across generations of panthers pre- and post-genetic rescue. The SE values are reflective of the combined sample within each cohort, since *q*-values presented are proportions of that sample. These same data are presented in **Extended Data Fig. 1**.

Cohort	Timeframe	n	Canonical	Admixed	Mean Canonical *q*-Value	Mean Admixed *q*-value	SE
Pre1	<1986	33	13	20	0.741	0.259	0.050
Pre2	1986-1995	55	38	17	0.849	0.151	0.028
Post1	1996-2005	167	35	132	0.557	0.443	0.023
Post2	2006-2015	237	2	235	0.479	0.521	0.012
Post3	2016-2020	55	1	54	0.538	0.462	0.022

Visualization of two cluster model of genetic population structure for all Florida panthers (including kittens)

The results of the STRUCTURE clustering analysis of ancestry that included all panthers (n = 904) is presented below in Fig A2.1. Similar to our analysis in the main text on a subset of data that included only adults and subadults, our analysis including kittens supported a two-cluster model (ΔK = 1786 at K = 2, Ln likelihood $_{(K=2)}$ = -31951, SD = 0.8) from which we used *q*-values (proportion of an individual's genome [i.e., ancestry] originating from a cluster in *K*) to assign panthers as either canonical (≥90% pre-introgression ancestry) or admixed (<90% pre-introgression ancestry). The homogeneous nature of ancestry in panthers is evident in Figure A2.1 panels A and B (Pre1 and Pre2 cohorts of panthers), a period when the population was at its nadir and comprised of highly inbred canonical panthers. Subsequently, after the release of 8 female pumas from Texas in 1995 (7 of 8 Texas females noted by stippled red bars at the beginning of panel C in both figures), we see significant increases in the levels of admixture in cohorts of panthers born in the Post1-3 periods (panels C-E).

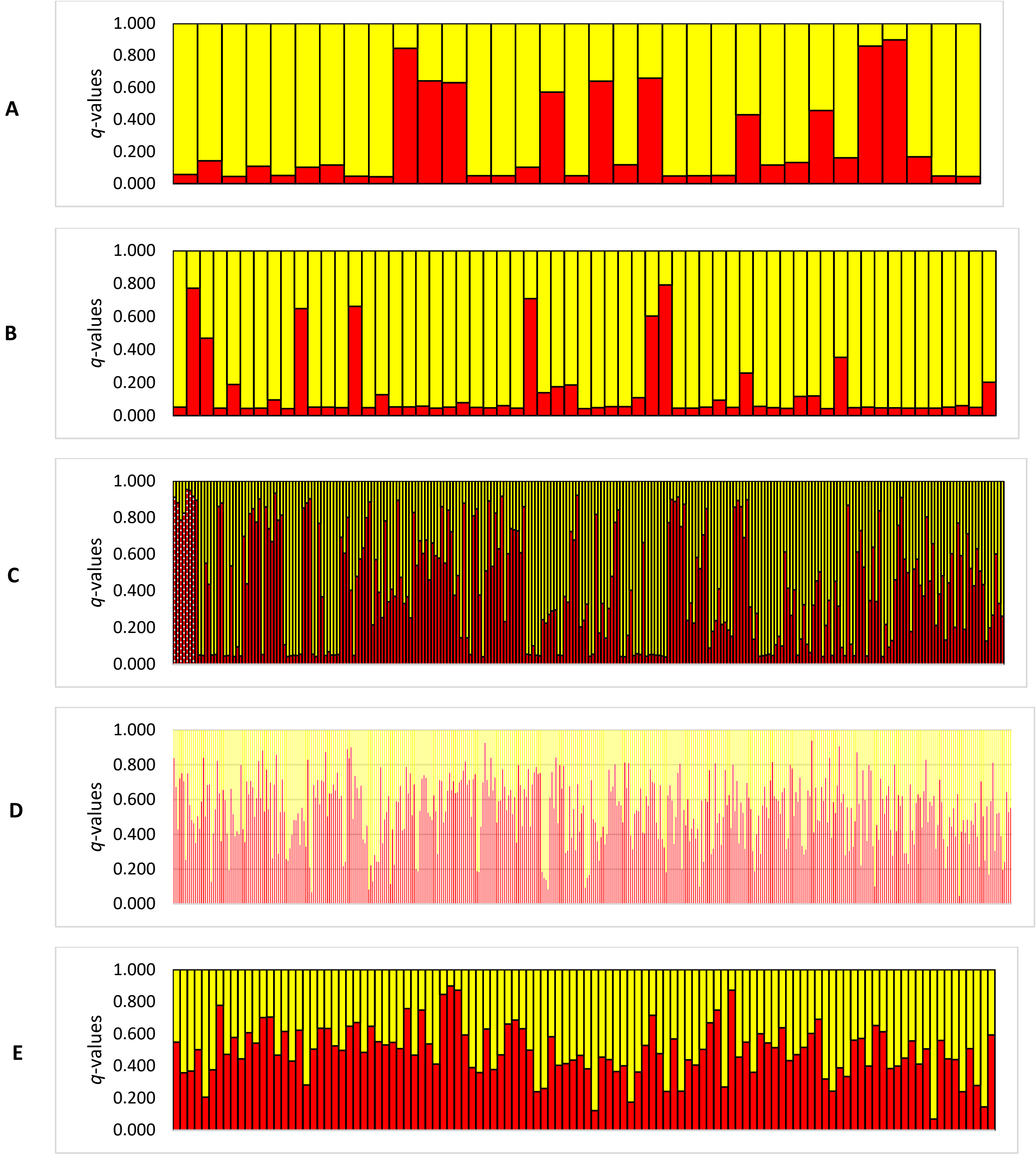

Figure A2.1 Results from STRUCTURE clustering analysis for Florida panthers (n = 904) with a *K* = 2, where the x-axis represents individual panthers in cohorts A) Pre1 (<1986), B) Pre2 (1986–1995), C) Post1 (1996–2005), D) Post2 (2006–2015), E) Post3 (2016–2020). Yellow indicates allele combinations associated with the canonical panthers and red indicates allele combinations associated with the admixture resulting from genetic rescue. The y-axis represents the percentage ancestry (*q*-values) for each ancestral group within each panther. This analysis was inclusive of kittens, subadult and adult Florida panthers. The first 7 individuals in panel C are the Texas female pumas released in 1995 (TX102 did not amplify); admixed bars are stippled red for clarity.

Three cluster model of genetic population structure

Although the K = 2 cluster model from our STRUCTURE analysis using microsatellite genotype data was most highly supported for our Florida panther dataset (ΔK = 1786 at K = 2; Ln likelihood $_{(K=2)}$ = -31951 sd = 0.8), we wanted to assess if the K = 3 cluster model (ΔK = 0.3424 at K = 3; Ln likelihood $_{(K=3)}$ = -31056 sd = 240) would effectively identify animals: 1) that are descendants of poorly documented releases of captive pumas into Everglades National Park in the 1950s and 1960s [19]; 2) that originate from other non-Florida populations. Previous work suggested that panthers with this "Everglades ancestry" were likely derived from a captive puma lineage from Central America [11,12,20] and the improved genetic fitness of these Everglades panthers was evident in several metrics that have been compared in previous research [11].

Results identified a canonical clade of Florida panthers, an admixed clade of Florida panthers, and a non-Florida clade. Of the 17 panthers that were either captured or collected in or near Everglades National Park in Miami-Dade County, the average non-Florida ancestry level was 0.315. Two of these panthers (FP039 and FP044) can be qualified as canonical with ancestry values for that clade of 0.982 and 0.992, respectively. The prevalence of the non-Florida ancestral group in the population has waned over time, especially since genetic rescue in 1995 (**Extended Data Fig. 3.**). The Texas and Western population of pumas from this model highlight how the non-Florida clade makes up a majority of the ancestry of all individuals in those populations (**Extended Data Fig. 3.**). By comparison, the Florida panther population has always been dominated by the canonical and admixed ancestral clades, regardless of whether the cohorts were present during the pre- or post-genetic rescue time periods. These findings provide additional support for the distinctness that the Florida panther population retains when compared to breeding populations of Western pumas.

Supplemental Information Appendix 3

Statistical analysis for correlates of inbreeding comparisons

Methods

We used binomial regression followed by post-hoc contrasts to quantify and compare the proportion of panthers in each cohort (Pre1, Pre2, Post1, Post2, and Post3) that exhibited kinked tails, cowlicks, atrial septal defects (ASD), and cryptorchidism. This cohort-specific analysis allowed us to assess the longevity of the benefits of genetic rescue via the prevalence of these traits in the panther population through time and across multiple generations pre- and post-genetic rescue. We assessed among-cohort differences in the mean percentage of abnormal sperm using a beta generalized linear regression model with a logit link function followed by post-hoc contrasts. For all post-hoc contrasts, we used the false discovery rate *P*-value adjustment method [21] and evaluated the significance of among-group differences using a threshold *P*-value of 0.05. Additionally, we assessed goodness-of-fit for the beta and binomial regression models using a simulation-based approach to residual analysis, implemented in the R package DHARMa. We used two-proportions Z-tests to compare the proportions of admixed and canonical panthers that exhibited kinked tails, cowlicks, ASD, and cryptorchidism. We conducted all statistical analyses in R v.4.2.1 [22] using the packages glmmTMB (binomial and beta regression models); [23], emmeans (post-hoc contrasts); [24], and DHARMa (goodness-of-fit) [25].

Results

Statistical comparisons between cohorts of panthers and ancestral categories are shown in Table A3.1. Overall, our binomial regression analyses with post-hoc comparisons revealed there were significantly higher proportions of panthers exhibiting kinks and cowlicks in the Pre1 and Pre2 cohorts compared to cohorts Post1, 2, and 3 (Table A3.1). Proportions of individuals within each cohort with cryptorchidism and ASDs decreased following genetic rescue, although these declines were not deemed statistically significant (Table A3.1). Although small sample size prevented us from statistically comparing Pre- and Post-rescue periods, the percentage of abnormal sperm became progressively lower during Post1, Post2, and Post3 periods, and percent abnormal sperm were 3.04, 5.06, and 6.03 times less likely in those periods compared to the single sample collected during Pre2. Overall, for the 5 generations since genetic rescue (Post1, 2 and 3), our results provide strong evidence that the population has benefited via declines in all these correlates of inbreeding.

The prevalence of these traits in panthers was different between canonical ($n = 85$) and admixed ($n = 433$) ancestral groups. Two-proportions Z-tests indicated that canonical panthers exhibited higher proportions of kinked tails ($Z = 111.53$ df = 1, $p<0.001$), cowlicks ($Z = 115.77$ df = 1, $p<0.001$), ASDs ($Z = 6.4073$ df = 1, $p=0.011$), and cryptorchidism ($Z = 59.078$ df = 1, $p<0.001$) compared to admixed panthers (Table A3.1). Beta regression results indicated that canonical panthers also had a higher percentage of structurally abnormal spermatozoa than admixed panthers (90% vs. 72.8%; Table A3.1). These results provide further evidence of the impact of inbreeding depression on the canonical clade of panthers that comprised the majority of the population prior to genetic rescue in 1995.

Table A3.1 Proportion of Florida panthers sampled from 1981–2021 that exhibited correlates of inbreeding depression for morphological and physiological traits. Comparisons are made according to the defined genetic ancestry of panthers and via the generations of panthers in cohorts that include periods of time pre- and post-genetic rescue. We used two-proportion Z-tests to determine significant comparisons (*, p <0.05) between ancestral categories. Different superscript letters indicate significant differences (p < 0.05; binomial regression analyses with post-hoc comparisons) between cohorts of panthers. The Post3 cohort includes data from 2021 for these correlates of inbreeding.

	Kinked Tail		Cowlick		Atrial Septal Defect		Cryptorchidism		Abnormal Sperm♦	
Ancestry	Proportion	n	Proportion	n	Proportion	n	Proportion	n	Average %	n
Canonical	0.882*	85	0.827*	81	0.207*	58	0.600*	50	90.0⋆	6
Admixed	0.270	433	0.213	413	0.085	294	0.110	228	72.8	38
Cohorts										
Pre1 (<1986)	0.852[A]	27	0.846[A]	26	0.273[A]	11	0.267[A]	15	-	-
Pre2 (1986-1995)	0.763[A]	59	0.754[A]	61	0.132[A]	38	0.553[AB]	38	97.0[A]	1
Post1 (1996-2005)	0.386[B]	171	0.340[B]	159	0.074[A]	108	0.226[AC]	93	79.0[A]	13
Post2 (2006-2015)	0.245[C]	269	0.171[C]	258	0.084[A]	190	0.090[AD]	134	71.4[A]	23
Post3 (2016-2021)	0.221[C]	86	0.189[C]	74	0.118[A]	76	0.067[AD]	45	69.4[A]	6

♦ Sperm quality data are from Penfold et al. [26].

⋆Mean percentage of abnormal sperm is higher in canonical than admixed panthers (beta regression: estimate (95% CI) =-1.0698 (-0.357, -1.78); p =0.003).

Supplemental Information Appendix 4
Statistical analysis using pairwise contrasts for measures of genetic diversity

Methods

We used GenAlex 6.5 [27,28] to determine the number of alleles (N_a), number of effective alleles ($N_{effective}$), observed heterozygosity (H_o), and expected heterozygosity (H_e). We used hp-rare [29] to estimate allelic richness (A_r) using the rarefaction technique to account for different sample sizes among groups of panthers and Western pumas. We used gamma or linear regression followed by post-hoc contrasts to assess among-group differences in these metrics separately for (a) panther ancestral groups and Western pumas, and (b) panther cohorts and Western pumas. For each model, we assessed goodness-of-fit using a simulation-based approach to residual analysis, implemented in the R package DHARMa goodness-of-fit; [25]. We conducted all statistical analyses and plotting in Program R using the packages glmmTMB model fitting; [23], emmeans post-hoc contrasts; [24], and DHARMa.

Results

Table A4.1 Metrics of genetic variation in ancestral groups and cohorts of Florida panthers pre- and post-genetic rescue sampled from 1981-2020 in Florida, USA. Values are means and standard errors calculated using genotype data collected from 16 microsatellite loci. Data from Western populations of puma are presented for comparative purposes. Metrics include number of alleles (N_a), number of effective alleles ($N_{effective}$), observed and expected heterozygosity (H_o, H_e), allelic richness (A_r), and individual heterozygosity (H_{ind}).

Ancestry	n	Na	$N_{effective}$	H_o	H_e	A_r	H_{ind}
Canonical	90	2.50 ± 0.20	1.61 ± 0.14	0.34 ± 0.06	0.31 ± 0.05	2.12 ± 0.15	0.37 ± 0.01
Admixed	462	6.13 ± 0.41	2.61 ± 0.23	0.55 ± 0.04	0.57 ± 0.04	4.38 ± 0.27	0.57 ± 0.01
Cohorts							
Pre1 (<1986)	33	4.75 ± 0.21	2.09 ± 0.16	0.45 ± 0.04	0.48 ± 0.04	4.30 ± 0.17	0.47 ± 0.04
Pre2 (1986-1995)	57	3.88 ± 0.26	1.79 ± 0.15	0.40 ± 0.05	0.39 ± 0.05	3.30 ± 0.18	0.43 ± 0.02
Post1 (1996-2005)	167	5.50 ± 0.38	2.46 ± 0.22	0.52 ± 0.04	0.55 ± 0.04	4.31 ± 0.27	0.55 ± 0.01
Post2 (2006-2015)	237	4.69 ± 0.38	2.56 ± 0.22	0.55 ± 0.04	0.56 ± 0.04	3.99 ± 0.27	0.57 ± 0.01
Post3 (2016-2020)	57	4.13 ± 0.33	2.40 ± 0.20	0.51 ± 0.04	0.54 ± 0.04	3.81 ± 0.25	0.53 ± 0.02
Western puma							
Colorado	23	4.44 ± 0.32	2.98 ± 0.20	0.62± 0.03	0.65 ± 0.02	4.40 ± 0.30	0.63 ± 0.03
Idaho	23	4.75 ± 0.40	3.31 ± 0.36	0.65 ± 0.04	0.65 ± 0.03	4.72 ± 0.40	0.64 ± 0.03
North Dakota	22	3.81 ± 0.38	2.60 ± 0.23	0.55 ± 0.05	0.57 ± 0.04	3.79 ± 0.37	0.56 ± 0.03
South Dakota	26	4.81 ± 0.37	2.89 ± 0.26	0.57 ± 0.04	0.61 ± 0.04	4.66 ± 0.35	0.57 ± 0.02
Texas	47	5.31 ± 0.27	3.20 ± 0.21	0.66 ± 0.03	0.66 ± 0.03	4.80 ± 0.20	0.66 ± 0.02

Table A. N_a -mean number of alleles statistics. Marginal means, pairwise contrasts, standard errors, p-values, degrees of freedom (df), t-ratios (Difference/SE), and 95% confidence intervals based on the gamma regression model used to assess among-group differences in the mean N_a at 16 microsatellite loci for canonical and admixed Florida panthers and Western puma populations. All values are on the natural log scale.

		Marginal means			
Group	Mean	SE	df	Lower	Upper
Admixed	1.812	0.072	104	1.669	1.960
Canonical	0.916	0.072	104	0.773	1.060
CO	1.490	0.072	104	1.347	1.630
ID	1.558	0.072	104	1.415	1.700
ND	1.338	0.072	104	1.195	1.480
SD	1.571	0.072	104	1.428	1.710
TX	1.670	0.072	104	1.527	1.810
		Post-hoc contrasts			
Contrast	Difference	SE	df	t-ratio	*p*-value
Admixed - Canonical	0.896	0.102	104	8.793	<.0001
Admixed - CO	0.322	0.102	104	3.162	0.005
Admixed - ID	0.254	0.102	104	2.495	0.030
Admixed - ND	0.474	0.102	104	4.652	<.0001
Admixed - SD	0.241	0.102	104	2.366	0.038
Admixed - TX	0.142	0.102	104	1.396	0.217
Canonical - CO	-0.574	0.102	104	-5.630	<.0001
Canonical - ID	-0.642	0.102	104	-6.298	<.0001
Canonical - ND	-0.422	0.102	104	-4.141	0.000
Canonical - SD	-0.655	0.102	104	-6.426	<.0001
Canonical - TX	-0.754	0.102	104	-7.396	<.0001
CO - ID	-0.068	0.102	104	-0.668	0.531
CO - ND	0.152	0.102	104	1.490	0.195
CO - SD	-0.081	0.102	104	-0.796	0.473
CO - TX	-0.180	0.102	104	-1.766	0.121
ID - ND	0.220	0.102	104	2.157	0.054
ID - SD	-0.013	0.102	104	-0.128	0.898
ID - TX	-0.112	0.102	104	-1.098	0.339
ND - SD	-0.233	0.102	104	-2.286	0.043
ND - TX	-0.332	0.102	104	-3.255	0.004
SD - TX	-0.099	0.102	104	-0.970	0.390

Table B. N_a -mean number of alleles statistics. Marginal means, pairwise contrasts, standard errors, p-values, degrees of freedom (df), t-ratios (Difference/SE), and 95% confidence intervals based on the gamma regression model used to assess among-group differences in the mean N_a at 16 microsatellite loci for pre- and post-rescue Florida panthers and Western puma populations. All values are on the natural log scale.

Marginal means					
Group	Mean	SE	df	Lower	Upper
Pre1	1.558	0.067	149	1.427	1.690
Pre2	1.355	0.067	149	1.223	1.486
Post1	1.705	0.067	149	1.573	1.836
Post2	1.545	0.067	149	1.413	1.676
Post3	1.417	0.067	149	1.286	1.549
CO	1.490	0.067	149	1.359	1.622
ID	1.558	0.067	149	1.427	1.690
ND	1.338	0.067	149	1.207	1.470
SD	1.571	0.067	149	1.440	1.703
TX	1.670	0.067	149	1.539	1.802
Post-hoc contrasts					
Contrast	Difference	SE	df	t-ratio	*p*-value
Pre1 - Pre2	0.204	0.094	149	2.163	0.103
Pre1 - Post1	-0.147	0.094	149	-1.558	0.260
Pre1 - Post2	0.013	0.094	149	0.141	0.910
Pre1 - Post3	0.141	0.094	149	1.499	0.266
Pre1 - CO	0.068	0.094	149	0.723	0.605
Pre1 - ID	0.000	0.094	149	0.000	1.000
Pre1 - ND	0.220	0.094	149	2.336	0.098
Pre1 - SD	-0.013	0.094	149	-0.139	0.910
Pre1 - TX	-0.112	0.094	149	-1.189	0.367
Pre2 - Post1	-0.350	0.094	149	-3.721	0.006
Pre2 - Post2	-0.190	0.094	149	-2.022	0.135
Pre2 - Post3	-0.063	0.094	149	-0.664	0.634
Pre2 - CO	-0.136	0.094	149	-1.440	0.285
Pre2 - ID	-0.204	0.094	149	-2.163	0.103
Pre2 - ND	0.016	0.094	149	0.173	0.910
Pre2 - SD	-0.217	0.094	149	-2.302	0.098
Pre2 - TX	-0.316	0.094	149	-3.352	0.011
Post1 - Post2	0.160	0.094	149	1.698	0.242
Post1 - Post3	0.288	0.094	149	3.057	0.024
Post1 - CO	0.215	0.094	149	2.281	0.098
Post1 - ID	0.147	0.094	149	1.558	0.260
Post1 - ND	0.366	0.094	149	3.894	0.006
Post1 - SD	0.134	0.094	149	1.419	0.285
Post1 - TX	0.035	0.094	149	0.369	0.844
Post2 - Post3	0.128	0.094	149	1.358	0.305
Post2 - CO	0.055	0.094	149	0.582	0.683
Post2 - ID	-0.013	0.094	149	-0.141	0.910
Post2 - ND	0.207	0.094	149	2.195	0.103
Post2 - SD	-0.026	0.094	149	-0.280	0.900
Post2 - TX	-0.125	0.094	149	-1.330	0.309
Post3 - CO	-0.073	0.094	149	-0.776	0.599
Post3 - ID	-0.141	0.094	149	-1.499	0.266
Post3 - ND	0.079	0.094	149	0.837	0.568
Post3 - SD	-0.154	0.094	149	-1.638	0.258
Post3 - TX	-0.253	0.094	149	-2.688	0.060
CO - ID	-0.068	0.094	149	-0.723	0.605
CO - ND	0.152	0.094	149	1.613	0.258
CO - SD	-0.081	0.094	149	-0.862	0.566
CO - TX	-0.180	0.094	149	-1.912	0.163
ID - ND	0.220	0.094	149	2.336	0.098
ID - SD	-0.013	0.094	149	-0.139	0.910
ID - TX	-0.112	0.094	149	-1.189	0.367
ND - SD	-0.233	0.094	149	-2.475	0.093
ND - TX	-0.332	0.094	149	-3.525	0.008
SD - TX	-0.099	0.094	149	-1.050	0.443

Table C. $N_{effective}$ -mean effective number of alleles statistics. Marginal means, pairwise contrasts, standard errors, p-values, degrees of freedom (df), t-ratios (Difference/SE), and 95% confidence intervals based on the linear regression model used to assess among-group differences in the mean $N_{effective}$ at 16 microsatellite loci for canonical and admixed Florida panthers and Western puma populations.

Marginal means					
Group	Mean	SE	df	Lower	Upper
Admixed	2.611	0.231	104	2.154	3.068
Canonical	1.612	0.231	104	1.155	2.069
CO	2.984	0.231	104	2.527	3.442
ID	3.305	0.231	104	2.848	3.762
ND	2.596	0.231	104	2.138	3.053
SD	2.893	0.231	104	2.435	3.350
TX	3.196	0.231	104	2.738	3.653

Post-hoc contrasts					
Contrast	Difference	SE	df	t-ratio	*p*-value
Canonical - Admixed	-0.999	0.326	104	-3.062	0.011
Canonical - CO	-1.372	0.326	104	-4.207	0.000
Canonical - ID	-1.693	0.326	104	-5.190	<.0001
Canonical - ND	-0.984	0.326	104	-3.016	0.011
Canonical - SD	-1.280	0.326	104	-3.926	0.001
Canonical - TX	-1.583	0.326	104	-4.854	<.0001
Admixed - CO	-0.373	0.326	104	-1.145	0.412
Admixed - ID	-0.694	0.326	104	-2.128	0.094
Admixed - ND	0.015	0.326	104	0.047	0.963
Admixed - SD	-0.282	0.326	104	-0.863	0.482
Admixed - TX	-0.585	0.326	104	-1.792	0.160
CO - ID	-0.321	0.326	104	-0.983	0.479
CO - ND	0.388	0.326	104	1.191	0.412
CO - SD	0.092	0.326	104	0.281	0.818
CO - TX	-0.211	0.326	104	-0.647	0.605
ID - ND	0.709	0.326	104	2.174	0.094
ID - SD	0.412	0.326	104	1.264	0.399
ID - TX	0.110	0.326	104	0.336	0.815
ND - SD	-0.297	0.326	104	-0.910	0.479
ND - TX	-0.600	0.326	104	-1.839	0.160
SD - TX	-0.303	0.326	104	-0.929	0.479

Table D. $N_{effective}$ -mean effective number of alleles statistics. Marginal means, pairwise contrasts, standard errors, p-values, degrees of freedom (df), t-ratios (Difference/SE), and 95% confidence intervals based on the linear regression model used to assess among-group differences in the mean $N_{effective}$ at 16 microsatellite loci for pre- and post-rescue Florida panthers and Western puma populations.

Marginal means					
Group	Mean	SE	df	Lower	Upper
Pre1	2.094	0.219	149	1.662	2.526
Pre2	1.791	0.219	149	1.359	2.224
Post1	2.456	0.219	149	2.023	2.888
Post2	2.557	0.219	149	2.124	2.989
Post3	2.403	0.219	149	1.971	2.836
CO	2.984	0.219	149	2.552	3.417
ID	3.305	0.219	149	2.873	3.738
ND	2.596	0.219	149	2.163	3.028
SD	2.893	0.219	149	2.460	3.325
TX	3.196	0.219	149	2.763	3.628
Post-hoc contrasts					
Contrast	Difference	SE	df	t-ratio	*p*-value
Pre1 - Pre2	0.303	0.310	149	0.977	0.424
Pre1 - Post1	-0.362	0.310	149	-1.169	0.366
Pre1 - Post2	-0.463	0.310	149	-1.495	0.247
Pre1 - Post3	-0.309	0.310	149	-0.999	0.424
Pre1 - CO	-0.890	0.310	149	-2.876	0.026
Pre1 - ID	-1.211	0.310	149	-3.913	0.002
Pre1 - ND	-0.502	0.310	149	-1.621	0.210
Pre1 - SD	-0.799	0.310	149	-2.580	0.043
Pre1 - TX	-1.102	0.310	149	-3.559	0.004
Pre2 - Post1	-0.664	0.310	149	-2.147	0.089
Pre2 - Post2	-0.765	0.310	149	-2.472	0.050
Pre2 - Post3	-0.612	0.310	149	-1.976	0.118
Pre2 - CO	-1.193	0.310	149	-3.854	0.002
Pre2 - ID	-1.514	0.310	149	-4.890	0.000
Pre2 - ND	-0.804	0.310	149	-2.599	0.043
Pre2 - SD	-1.101	0.310	149	-3.558	0.004
Pre2 - TX	-1.404	0.310	149	-4.536	0.000
Post1 - Post2	-0.101	0.310	149	-0.326	0.799
Post1 - Post3	0.053	0.310	149	0.170	0.885
Post1 - CO	-0.528	0.310	149	-1.707	0.184
Post1 - ID	-0.849	0.310	149	-2.744	0.034
Post1 - ND	-0.140	0.310	149	-0.452	0.733
Post1 - SD	-0.437	0.310	149	-1.411	0.277
Post1 - TX	-0.740	0.310	149	-2.390	0.054
Post2 - Post3	0.153	0.310	149	0.496	0.717
Post2 - CO	-0.428	0.310	149	-1.382	0.282
Post2 - ID	-0.748	0.310	149	-2.418	0.054
Post2 - ND	-0.039	0.310	149	-0.127	0.899
Post2 - SD	-0.336	0.310	149	-1.086	0.406
Post2 - TX	-0.639	0.310	149	-2.064	0.102
Post3 - CO	-0.581	0.310	149	-1.877	0.134
Post3 - ID	-0.902	0.310	149	-2.914	0.026
Post3 - ND	-0.193	0.310	149	-0.622	0.633
Post3 - SD	-0.489	0.310	149	-1.581	0.217
Post3 - TX	-0.792	0.310	149	-2.560	0.043
CO - ID	-0.321	0.310	149	-1.036	0.424
CO - ND	0.389	0.310	149	1.255	0.328
CO - SD	0.092	0.310	149	0.296	0.803
CO - TX	-0.211	0.310	149	-0.682	0.603
ID - ND	0.709	0.310	149	2.292	0.066
ID - SD	0.412	0.310	149	1.333	0.297
ID - TX	0.110	0.310	149	0.354	0.795
ND - SD	-0.297	0.310	149	-0.959	0.424
ND - TX	-0.600	0.310	149	-1.938	0.123
SD - TX	-0.303	0.310	149	-0.979	0.424

Table E. A_r-mean allelic richness statistics. Marginal means, pairwise contrasts, standard errors, p-values, degrees of freedom (df), t-ratios (Difference/SE), and 95% confidence intervals based on the gamma regression model used to assess among-group differences in mean A_r at 16 microsatellite loci for canonical and admixed Florida panthers and Western puma populations. All values are on the natural log scale.

Marginal means					
Group	Mean	SE	df	Lower	Upper
Admixed	1.477	0.068	104	1.342	1.611
Canonical	0.750	0.068	104	0.616	0.884
CO	1.480	0.068	104	1.346	1.615
ID	1.551	0.068	104	1.417	1.685
ND	1.333	0.068	104	1.199	1.467
SD	1.539	0.068	104	1.405	1.674
TX	1.569	0.068	104	1.434	1.703
Post-hoc contrasts					
Contrast	Difference	SE	df	t-ratio	*p*-value
Canonical - Admixed	-0.727	0.096	104	-7.589	<.0001
Canonical - CO	-0.730	0.096	104	-7.630	<.0001
Canonical - ID	-0.801	0.096	104	-8.365	<.0001
Canonical - ND	-0.583	0.096	104	-6.089	<.0001
Canonical - SD	-0.789	0.096	104	-8.245	<.0001
Canonical - TX	-0.819	0.096	104	-8.551	<.0001
Admixed - CO	-0.004	0.096	104	-0.040	0.968
Admixed - ID	-0.074	0.096	104	-0.776	0.650
Admixed - ND	0.144	0.096	104	1.501	0.261
Admixed - SD	-0.063	0.096	104	-0.656	0.667
Admixed - TX	-0.092	0.096	104	-0.962	0.580
CO - ID	-0.070	0.096	104	-0.735	0.650
CO - ND	0.148	0.096	104	1.541	0.261
CO - SD	-0.059	0.096	104	-0.615	0.667
CO - TX	-0.088	0.096	104	-0.921	0.580
ID - ND	0.218	0.096	104	2.276	0.065
ID - SD	0.011	0.096	104	0.120	0.950
ID - TX	-0.018	0.096	104	-0.186	0.943
ND - SD	-0.206	0.096	104	-2.156	0.078
ND - TX	-0.236	0.096	104	-2.462	0.046
SD - TX	-0.029	0.096	104	-0.306	0.887

Table F. A_r-mean allelic richness statistics. Marginal means, pairwise contrasts, standard errors, p-values, degrees of freedom (df), t-ratios (Difference/SE), and 95% confidence intervals based on the gamma regression model used to assess among-group differences in the mean A_r at 16 microsatellite loci for pre- and post-rescue Florida panthers and Western puma populations. All values are on the natural log scale.

Marginal means					
Group	Mean	SE	df	Lower	Upper
Pre1	1.458	0.062	149	1.335	1.580
Pre2	1.195	0.062	149	1.073	1.317
Post1	1.461	0.062	149	1.339	1.584
Post2	1.383	0.062	149	1.261	1.506
Post3	1.337	0.062	149	1.215	1.459
CO	1.480	0.062	149	1.358	1.603
ID	1.551	0.062	149	1.428	1.673
ND	1.333	0.062	149	1.211	1.455
SD	1.539	0.062	149	1.417	1.662
TX	1.569	0.062	149	1.446	1.691
Post-hoc contrasts					
Contrast	Difference	SE	df	t-ratio	*p*-value
Pre1 - Pre2	0.263	0.088	149	2.999	0.024
Pre1 - Post1	-0.004	0.088	149	-0.040	0.968
Pre1 - Post2	0.074	0.088	149	0.847	0.527
Pre1 - Post3	0.121	0.088	149	1.378	0.319
Pre1 - CO	-0.023	0.088	149	-0.261	0.894
Pre1 - ID	-0.093	0.088	149	-1.064	0.465
Pre1 - ND	0.125	0.088	149	1.423	0.310
Pre1 - SD	-0.082	0.088	149	-0.933	0.512
Pre1 - TX	-0.111	0.088	149	-1.267	0.373
Pre2 - Post1	-0.266	0.088	149	-3.039	0.024
Pre2 - Post2	-0.188	0.088	149	-2.152	0.114
Pre2 - Post3	-0.142	0.088	149	-1.622	0.253
Pre2 - CO	-0.286	0.088	149	-3.260	0.016
Pre2 - ID	-0.356	0.088	149	-4.063	0.002
Pre2 - ND	-0.138	0.088	149	-1.576	0.264
Pre2 - SD	-0.344	0.088	149	-3.932	0.002
Pre2 - TX	-0.374	0.088	149	-4.267	0.002
Post1 - Post2	0.078	0.088	149	0.888	0.513
Post1 - Post3	0.124	0.088	149	1.418	0.310
Post1 - CO	-0.019	0.088	149	-0.220	0.899
Post1 - ID	-0.090	0.088	149	-1.024	0.474
Post1 - ND	0.128	0.088	149	1.464	0.310
Post1 - SD	-0.078	0.088	149	-0.893	0.513
Post1 - TX	-0.107	0.088	149	-1.227	0.384
Post2 - Post3	0.046	0.088	149	0.530	0.707
Post2 - CO	-0.097	0.088	149	-1.108	0.449
Post2 - ID	-0.167	0.088	149	-1.912	0.174
Post2 - ND	0.050	0.088	149	0.576	0.688
Post2 - SD	-0.156	0.088	149	-1.780	0.217
Post2 - TX	-0.185	0.088	149	-2.115	0.116
Post3 - CO	-0.144	0.088	149	-1.638	0.253
Post3 - ID	-0.214	0.088	149	-2.442	0.071
Post3 - ND	0.004	0.088	149	0.046	0.968
Post3 - SD	-0.202	0.088	149	-2.311	0.083
Post3 - TX	-0.232	0.088	149	-2.645	0.051
CO - ID	-0.070	0.088	149	-0.803	0.544
CO - ND	0.148	0.088	149	1.684	0.250
CO - SD	-0.059	0.088	149	-0.672	0.628
CO - TX	-0.088	0.088	149	-1.007	0.474
ID - ND	0.218	0.088	149	2.488	0.070
ID - SD	0.011	0.088	149	0.131	0.938
ID - TX	-0.018	0.088	149	-0.203	0.899
ND - SD	-0.206	0.088	149	-2.356	0.081
ND - TX	-0.236	0.088	149	-2.691	0.051
SD - TX	-0.029	0.088	149	-0.334	0.852

Table G. H_o-mean observed heterozygosity statistics. Marginal means, pairwise contrasts, standard errors, p-values, degrees of freedom (df), t-ratios (Difference/SE), and 95% confidence intervals based on the linear regression model used to assess among-group differences in the mean H_o at 16 microsatellite loci for canonical and admixed Florida panthers and Western puma populations.

Marginal means					
Group	Mean	SE	df	Lower	Upper
Admixed	0.551	0.034	98	0.483	0.618
Canonical	0.339	0.058	98	0.224	0.455
CO	0.621	0.031	98	0.560	0.682
ID	0.646	0.041	98	0.565	0.727
ND	0.554	0.052	98	0.451	0.657
SD	0.570	0.041	98	0.488	0.651
TX	0.661	0.031	98	0.600	0.722
Post-hoc contrasts					
Contrast	Difference	SE	df	t-ratio	*p*-value
Canonical - Admixed	-0.212	0.067	98	-3.143	0.009
Canonical - CO	-0.282	0.066	98	-4.284	0.000
Canonical - ID	-0.307	0.071	98	-4.315	0.000
Canonical - ND	-0.215	0.078	98	-2.755	0.025
Canonical - SD	-0.230	0.071	98	-3.236	0.009
Canonical - TX	-0.322	0.066	98	-4.888	0.000
Admixed - CO	-0.070	0.046	98	-1.535	0.244
Admixed - ID	-0.095	0.053	98	-1.792	0.168
Admixed - ND	-0.003	0.062	98	-0.051	0.959
Admixed - SD	-0.019	0.053	98	-0.353	0.846
Admixed - TX	-0.110	0.046	98	-2.401	0.055
CO - ID	-0.025	0.051	98	-0.487	0.775
CO - ND	0.067	0.060	98	1.112	0.403
CO - SD	0.052	0.051	98	1.003	0.445
CO - TX	-0.040	0.044	98	-0.910	0.479
ID - ND	0.092	0.066	98	1.393	0.292
ID - SD	0.077	0.058	98	1.319	0.308
ID - TX	-0.015	0.051	98	-0.286	0.855
ND - SD	-0.016	0.066	98	-0.236	0.855
ND - TX	-0.107	0.060	98	-1.769	0.168
SD - TX	-0.091	0.051	98	-1.776	0.168

Table H. H_o-mean observed heterozygosity statistics. Marginal means, pairwise contrasts, standard errors, p-values, degrees of freedom (df), t-ratios (Difference/SE), and 95% confidence intervals based on the linear regression model used to assess among-group differences in the mean H_o at 16 microsatellite loci for pre- and post-rescue Florida panthers and Western puma populations.

Marginal means					
Group	Mean	SE	df	Lower	Upper
Pre1	0.450	0.041	149	0.369	0.530
Pre2	0.396	0.041	149	0.316	0.476
Post1	0.524	0.041	149	0.444	0.604
Post2	0.550	0.041	149	0.470	0.630
Post3	0.509	0.041	149	0.429	0.589
CO	0.621	0.041	149	0.541	0.701
ID	0.646	0.041	149	0.566	0.726
ND	0.554	0.041	149	0.474	0.634
SD	0.570	0.041	149	0.489	0.650
TX	0.661	0.041	149	0.581	0.741
Post-hoc contrasts					
Contrast	Difference	SE	df	t-ratio	*p*-value
Pre1 - Pre2	0.054	0.057	149	0.933	0.496
Pre1 - Post1	-0.074	0.057	149	-1.296	0.329
Pre1 - Post2	-0.100	0.057	149	-1.751	0.176
Pre1 - Post3	-0.060	0.057	149	-1.042	0.434
Pre1 - CO	-0.172	0.057	149	-2.992	0.021
Pre1 - ID	-0.197	0.057	149	-3.428	0.007
Pre1 - ND	-0.104	0.057	149	-1.821	0.159
Pre1 - SD	-0.120	0.057	149	-2.093	0.114
Pre1 - TX	-0.211	0.057	149	-3.684	0.004
Pre2 - Post1	-0.128	0.057	149	-2.229	0.095
Pre2 - Post2	-0.154	0.057	149	-2.684	0.041
Pre2 - Post3	-0.113	0.057	149	-1.975	0.138
Pre2 - CO	-0.225	0.057	149	-3.925	0.002
Pre2 - ID	-0.250	0.057	149	-4.361	0.001
Pre2 - ND	-0.158	0.057	149	-2.754	0.037
Pre2 - SD	-0.174	0.057	149	-3.026	0.021
Pre2 - TX	-0.265	0.057	149	-4.617	0.000
Post1 - Post2	-0.026	0.057	149	-0.456	0.747
Post1 - Post3	0.015	0.057	149	0.254	0.818
Post1 - CO	-0.097	0.057	149	-1.697	0.187
Post1 - ID	-0.122	0.057	149	-2.133	0.111
Post1 - ND	-0.030	0.057	149	-0.525	0.711
Post1 - SD	-0.046	0.057	149	-0.798	0.562
Post1 - TX	-0.137	0.057	149	-2.389	0.069
Post2 - Post3	0.041	0.057	149	0.709	0.596
Post2 - CO	-0.071	0.057	149	-1.241	0.348
Post2 - ID	-0.096	0.057	149	-1.677	0.187
Post2 - ND	-0.004	0.057	149	-0.070	0.945
Post2 - SD	-0.020	0.057	149	-0.342	0.804
Post2 - TX	-0.111	0.057	149	-1.933	0.138
Post3 - CO	-0.112	0.057	149	-1.951	0.138
Post3 - ID	-0.137	0.057	149	-2.387	0.069
Post3 - ND	-0.045	0.057	149	-0.779	0.562
Post3 - SD	-0.060	0.057	149	-1.052	0.434
Post3 - TX	-0.152	0.057	149	-2.643	0.041
CO - ID	-0.025	0.057	149	-0.436	0.747
CO - ND	0.067	0.057	149	1.171	0.378
CO - SD	0.052	0.057	149	0.899	0.505
CO - TX	-0.040	0.057	149	-0.692	0.596
ID - ND	0.092	0.057	149	1.607	0.205
ID - SD	0.077	0.057	149	1.335	0.318
ID - TX	-0.015	0.057	149	-0.256	0.818
ND - SD	-0.016	0.057	149	-0.272	0.818
ND - TX	-0.107	0.057	149	-1.863	0.152
SD - TX	-0.091	0.057	149	-1.591	0.205

Table I. H_e-mean expected heterozygosity statistics. Marginal means, pairwise contrasts, standard errors, p-values, degrees of freedom (df), t-ratios (Difference/SE), and 95% confidence intervals based on the linear regression model used to assess among-group differences in mean H_e at 16 microsatellite loci for canonical and admixed Florida panthers and Western puma populations.

Marginal means					
Group	Mean	SE	df	Lower	Upper
Admixed	0.574	0.035	98	0.504	0.644
Canonical	0.312	0.053	98	0.208	0.416
CO	0.645	0.021	98	0.604	0.686
ID	0.648	0.033	98	0.582	0.714
ND	0.570	0.037	98	0.497	0.643
SD	0.607	0.039	98	0.529	0.684
TX	0.660	0.030	98	0.601	0.719
Post-hoc contrasts					
Contrast	Difference	SE	df	t-ratio	*p*-value
Canonical - Admixed	-0.262	0.063	98	-4.143	0.000
Canonical - CO	-0.333	0.057	98	-5.886	<.0001
Canonical - ID	-0.336	0.062	98	-5.395	<.0001
Canonical - ND	-0.258	0.064	98	-4.019	0.000
Canonical - SD	-0.295	0.066	98	-4.493	0.000
Canonical - TX	-0.348	0.060	98	-5.764	<.0001
Admixed - CO	-0.071	0.041	98	-1.737	0.180
Admixed - ID	-0.074	0.048	98	-1.528	0.227
Admixed - ND	0.004	0.051	98	0.085	0.937
Admixed - SD	-0.033	0.053	98	-0.620	0.663
Admixed - TX	-0.086	0.046	98	-1.868	0.170
CO - ID	-0.003	0.039	98	-0.080	0.937
CO - ND	0.075	0.042	98	1.783	0.180
CO - SD	0.038	0.044	98	0.862	0.586
CO - TX	-0.015	0.036	98	-0.411	0.795
ID - ND	0.078	0.050	98	1.580	0.224
ID - SD	0.041	0.051	98	0.804	0.593
ID - TX	-0.012	0.045	98	-0.264	0.876
ND - SD	-0.037	0.054	98	-0.688	0.647
ND - TX	-0.090	0.047	98	-1.912	0.170
SD - TX	-0.053	0.049	98	-1.082	0.455

Table J. H_e-mean expected heterozygosity statistics. Marginal means, pairwise contrasts, standard errors, p-values, degrees of freedom (df), t-ratios (Difference/SE), and 95% confidence intervals based on the linear regression model used to assess among-group differences in the mean H_e at 16 microsatellite loci for pre- and post-rescue Florida panthers and Western puma populations.

		Marginal means			
Group	Mean	SE	df	Lower	Upper
Pre1	0.479	0.039	140	0.402	0.556
Pre2	0.386	0.045	140	0.297	0.475
Post1	0.547	0.037	140	0.473	0.621
Post2	0.563	0.037	140	0.490	0.636
Post3	0.535	0.039	140	0.457	0.613
CO	0.645	0.021	140	0.604	0.686
ID	0.648	0.033	140	0.582	0.714
ND	0.570	0.037	140	0.497	0.642
SD	0.607	0.039	140	0.529	0.684
TX	0.660	0.030	140	0.601	0.718
		Post-hoc contrasts			
Contrast	Difference	SE	df	t-ratio	*p*-value
Pre1 - Pre2	0.093	0.059	140	1.559	0.202
Pre1 - Post1	-0.068	0.054	140	-1.266	0.322
Pre1 - Post2	-0.084	0.054	140	-1.566	0.202
Pre1 - Post3	-0.056	0.055	140	-1.018	0.437
Pre1 - CO	-0.166	0.044	140	-3.764	0.002
Pre1 - ID	-0.169	0.051	140	-3.302	0.008
Pre1 - ND	-0.091	0.054	140	-1.702	0.171
Pre1 - SD	-0.128	0.055	140	-2.317	0.065
Pre1 - TX	-0.181	0.049	140	-3.703	0.002
Pre2 - Post1	-0.161	0.058	140	-2.760	0.030
Pre2 - Post2	-0.177	0.058	140	-3.040	0.014
Pre2 - Post3	-0.149	0.060	140	-2.497	0.051
Pre2 - CO	-0.259	0.049	140	-5.235	<.0001
Pre2 - ID	-0.262	0.056	140	-4.686	0.000
Pre2 - ND	-0.184	0.058	140	-3.170	0.011
Pre2 - SD	-0.221	0.060	140	-3.704	0.002
Pre2 - TX	-0.274	0.054	140	-5.094	<.0001
Post1 - Post2	-0.016	0.052	140	-0.302	0.838
Post1 - Post3	0.012	0.054	140	0.219	0.865
Post1 - CO	-0.098	0.043	140	-2.298	0.065
Post1 - ID	-0.101	0.050	140	-2.023	0.107
Post1 - ND	-0.023	0.052	140	-0.437	0.765
Post1 - SD	-0.060	0.054	140	-1.106	0.406
Post1 - TX	-0.113	0.048	140	-2.374	0.061
Post2 - Post3	0.028	0.054	140	0.513	0.721
Post2 - CO	-0.082	0.042	140	-1.939	0.123
Post2 - ID	-0.085	0.050	140	-1.714	0.171
Post2 - ND	-0.007	0.052	140	-0.134	0.914
Post2 - SD	-0.044	0.054	140	-0.816	0.543
Post2 - TX	-0.097	0.047	140	-2.051	0.105
Post3 - CO	-0.110	0.045	140	-2.469	0.051
Post3 - ID	-0.113	0.052	140	-2.191	0.080
Post3 - ND	-0.035	0.054	140	-0.645	0.633
Post3 - SD	-0.072	0.056	140	-1.290	0.320
Post3 - TX	-0.125	0.049	140	-2.534	0.051
CO - ID	-0.003	0.039	140	-0.080	0.937
CO - ND	0.075	0.042	140	1.783	0.157
CO - SD	0.038	0.044	140	0.862	0.532
CO - TX	-0.015	0.036	140	-0.411	0.767
ID - ND	0.078	0.050	140	1.580	0.202
ID - SD	0.041	0.051	140	0.804	0.543
ID - TX	-0.012	0.045	140	-0.264	0.849
ND - SD	-0.037	0.054	140	-0.688	0.615
ND - TX	-0.090	0.047	140	-1.912	0.124
SD - TX	-0.053	0.049	140	-1.082	0.408

Supplemental Information Appendix 5

Comparing differences in individual heterozygosity (H_{ind}) among groups and assessment of a heterozygosity-fitness correlation between levels of H_{ind} and expression of correlates of inbreeding

Methods

Inbreeding depression in wild populations is commonly assessed using heterozygosity–fitness correlations (HFC), although their application in endangered populations is less common [30]. We estimated the heterozygosity of each individual panther by first calculating homozygosity by loci (HL), which varies between 0 (all loci heterozygous) and 1 all loci homozygous; [31] using the Rhh package [32] in Program R. We then calculated individual heterozygosity (H_{ind}) for each panther as 1 – HL see [33], thereby creating a scale where values increase as an individual approaches having a genotype that is completely heterozygous [34]. We used linear regression to quantify among-group differences in mean H_{ind} separately for (a) panther ancestral groups and Western pumas, and (b) panther cohorts and Western pumas. For our HFC analysis, we then used logistic regression to quantify among-group differences in the probability of expressing a correlate of inbreeding and to estimate the influence of H_{ind}, a continuous predictor variable, on the probability of trait expression. Here, the response variable was 1 if a given correlate of inbreeding was expressed and 0 otherwise. For each trait, the model included the additive effects of H_{ind} and either a panther ancestral group or cohort categorical predictor. Because of data limitations, we did not include a H_{ind} × panther ancestral group/panther cohort interaction term in the model. Following model fitting, we conducted a goodness-of-fit assessment for each model using the DHARMa package and evaluated predicted performance for logistic regression models by calculating Brier scores, where scores closer to zero indicated a better-predicting model [35]. We conducted all statistical analyses in Program R using packages glmmTMB, emmeans, and rms Brier and AUC scores; [36].

Results

Comparisons of individual heterozygosity (H_{ind}) values between ancestral groups and Western puma revealed significant improvements when comparing admixed to canonical panthers (Table A5.1). The H_{ind} values for Western pumas compared to admixed panthers were not significantly different, except when compared to the Texas population, whose H_{ind} values were on average higher. The H_{ind} values for canonical panthers were significantly lower than all values for the Western puma populations (Table A5.1). Comparisons of H_{ind} values between cohorts of panthers born pre- and post-genetic rescue and Western puma (Table A5.2) followed similar trends to the ancestry comparison, with some exceptions. The pre-genetic rescue cohorts (Pre1 and Pre2) did not have H_{ind} values that were significantly different from each other; post-genetic rescue (Post 1, 2, and 3) cohort H_{ind} values were also not significantly different from each other (Table A5.2). The H_{ind} values post-genetic rescue cohorts were significantly higher on average when compared to Pre1 and Pre2, except for Pre1 being not significantly different from the H_{ind} values for Post3. The general trend for comparing Pre1 and Pre2 to Western pumas showed significantly reduced H_{ind} values on average for the pre-genetic rescue panthers. Post-genetic rescue panthers were on average no different than Western pumas H_{ind}, except for Post1 being significantly lower than H_{ind} values for Idaho and Texas (Table A5.2). Figure A5.1 presents a visual depiction of the differences in H_{ind} for all groups. Results for the logistic regression analysis of HFC between H_{ind} and the probability of expressing a correlate of inbreeding are presented in Fig. 4 in the main text.

Table A5.1 Marginal means, pairwise contrasts, standard errors, p-values, degrees of freedom (df), t-ratios (Difference/SE), and 95% confidence intervals based on the linear regression model used to assess among-group differences in the mean individual heterozygosity (H_{ind}) at 16 microsatellite loci for canonical and admixed Florida panthers and Western puma populations.

Marginal means					
Group	Mean	SE	df	Lower	Upper
Admixed	0.574	0.007	686	0.561	0.587
Canonical	0.369	0.015	686	0.339	0.399
CO	0.626	0.030	686	0.567	0.685
ID	0.644	0.030	686	0.585	0.703
ND	0.560	0.031	686	0.499	0.620
SD	0.566	0.028	686	0.511	0.622
TX	0.659	0.021	686	0.618	0.700
Post-hoc contrasts					
Contrast	Difference	SE	df	t-ratio	p-value
Admixed - Canonical	0.205	0.017	686	12.374	<.0001
Admixed - CO	-0.052	0.031	686	-1.685	0.149
Admixed - ID	-0.069	0.031	686	-2.260	0.051
Admixed - ND	0.015	0.031	686	0.462	0.749
Admixed - SD	0.008	0.029	686	0.268	0.828
Admixed - TX	-0.085	0.022	686	-3.852	0.000
Canonical - CO	-0.257	0.034	686	-7.643	<.0001
Canonical - ID	-0.275	0.034	686	-8.168	<.0001
Canonical - ND	-0.191	0.034	686	-5.571	<.0001
Canonical - SD	-0.197	0.032	686	-6.160	<.0001
Canonical - TX	-0.290	0.026	686	-11.199	<.0001
CO - ID	-0.018	0.042	686	-0.416	0.749
CO - ND	0.066	0.043	686	1.545	0.184
CO - SD	0.060	0.041	686	1.447	0.208
CO - TX	-0.033	0.037	686	-0.903	0.482
ID - ND	0.084	0.043	686	1.957	0.097
ID - SD	0.077	0.041	686	1.875	0.107
ID - TX	-0.015	0.037	686	-0.421	0.749
ND - SD	-0.007	0.042	686	-0.161	0.872
ND - TX	-0.099	0.037	686	-2.673	0.020
SD - TX	-0.093	0.035	686	-2.634	0.020

Table A5.2 Marginal means, pairwise contrasts, standard errors, *p*-values, degrees of freedom (df), t-ratios (Difference/SE), and 95% confidence intervals based on the linear regression model used to assess among-group differences in mean individual heterozygosity (H_{ind}) at 16 microsatellite loci for pre- and post-rescue Florida panthers and Western puma populations.

Marginal means					
Group	Mean	SE	df	Lower	Upper
Pre1	0.471	0.027	683	0.418	0.523
Pre2	0.425	0.020	683	0.385	0.465
Post1	0.551	0.012	683	0.528	0.575
Post2	0.573	0.010	683	0.553	0.593
Post3	0.532	0.020	683	0.493	0.572
CO	0.626	0.032	683	0.563	0.689
ID	0.644	0.032	683	0.581	0.707
ND	0.560	0.033	683	0.495	0.624
SD	0.566	0.030	683	0.507	0.626
TX	0.659	0.022	683	0.615	0.703
Post-hoc contrasts					
Contrast	Difference	SE	df	t-ratio	*p*-value
Pre1 - Pre2	0.204	0.094	149	2.163	0.103
Pre1 - Post1	-0.147	0.094	149	-1.558	0.260
Pre1 - Post2	0.013	0.094	149	0.141	0.910
Pre1 - Post3	0.141	0.094	149	1.499	0.266
Pre1 - CO	0.068	0.094	149	0.723	0.605
Pre1 - ID	0.000	0.094	149	0.000	1.000
Pre1 - ND	0.220	0.094	149	2.336	0.098
Pre1 - SD	-0.013	0.094	149	-0.139	0.910
Pre1 - TX	-0.112	0.094	149	-1.189	0.367
Pre2 - Post1	-0.350	0.094	149	-3.721	0.006
Pre2 - Post2	-0.190	0.094	149	-2.022	0.135
Pre2 - Post3	-0.063	0.094	149	-0.664	0.634
Pre2 - CO	-0.136	0.094	149	-1.440	0.285
Pre2 - ID	-0.204	0.094	149	-2.163	0.103
Pre2 - ND	0.016	0.094	149	0.173	0.910
Pre2 - SD	-0.217	0.094	149	-2.302	0.098
Pre2 - TX	-0.316	0.094	149	-3.352	0.011
Post1 - Post2	0.160	0.094	149	1.698	0.242
Post1 - Post3	0.288	0.094	149	3.057	0.024
Post1 - CO	0.215	0.094	149	2.281	0.098
Post1 - ID	0.147	0.094	149	1.558	0.260
Post1 - ND	0.366	0.094	149	3.894	0.006
Post1 - SD	0.134	0.094	149	1.419	0.285
Post1 - TX	0.035	0.094	149	0.369	0.844
Post2 - Post3	0.128	0.094	149	1.358	0.305
Post2 - CO	0.055	0.094	149	0.582	0.683
Post2 - ID	-0.013	0.094	149	-0.141	0.910
Post2 - ND	0.207	0.094	149	2.195	0.103
Post2 - SD	-0.026	0.094	149	-0.280	0.900
Post2 - TX	-0.125	0.094	149	-1.330	0.309
Post3 - CO	-0.073	0.094	149	-0.776	0.599
Post3 - ID	-0.141	0.094	149	-1.499	0.266
Post3 - ND	0.079	0.094	149	0.837	0.568
Post3 - SD	-0.154	0.094	149	-1.638	0.258
Post3 - TX	-0.253	0.094	149	-2.688	0.060
CO - ID	-0.068	0.094	149	-0.723	0.605
CO - ND	0.152	0.094	149	1.613	0.258
CO - SD	-0.081	0.094	149	-0.862	0.566
CO - TX	-0.180	0.094	149	-1.912	0.163
ID - ND	0.220	0.094	149	2.336	0.098
ID - SD	-0.013	0.094	149	-0.139	0.910
ID - TX	-0.112	0.094	149	-1.189	0.367
ND - SD	-0.233	0.094	149	-2.475	0.093
ND - TX	-0.332	0.094	149	-3.525	0.008
SD - TX	-0.099	0.094	149	-1.050	0.443

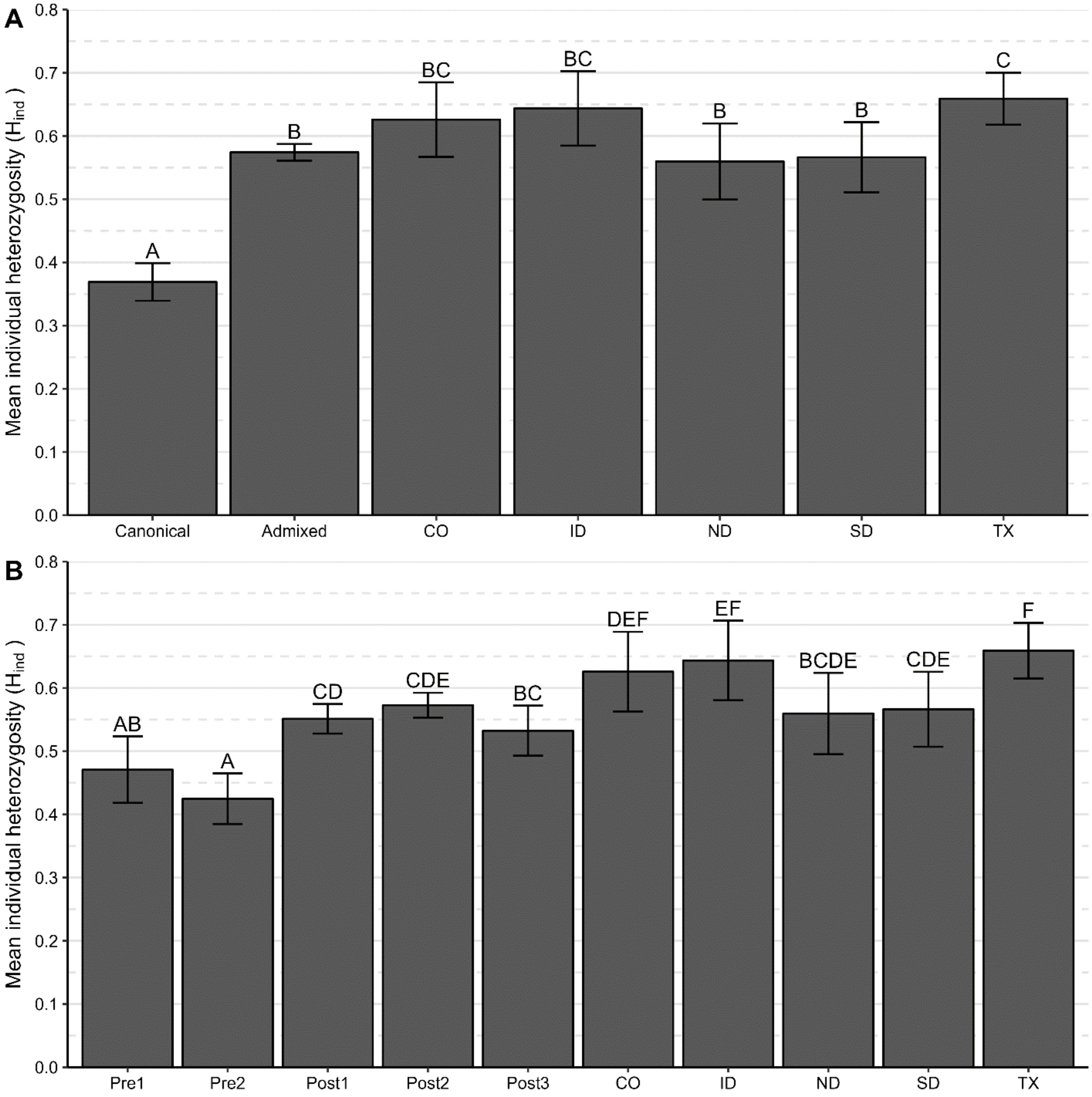

Figure A5.1 Visual representations of comparisons of mean H_{ind} values for A) ancestral groups of Florida panthers and B) cohorts of Florida panthers born pre- and post-genetic rescue compared to mean H_{ind} for Western pumas. Significant differences in pairwise contrasts between groups are denoted by differing letters above the bars.

Supplemental Information Appendix 6

Population size estimate using panther motor vehicle collision mortality data

We revised the model of McClintock et al.[37] with additional panther road mortality and radiocollar monitoring data collected from 2013–2018 to estimate the annual, range-wide, size of the panther population for an 18-year period in the post-genetic rescue era (2000–2018). Data collection and statistical modeling methods are described in detail in McClintock et al. [37]. To allow for more flexible (and potentially more parsimonious) population trend models, the revised analysis included multiple spline-based models of abundance. Relative to the polynomial trend models originally used by McClintock et al. [37], we found some of the annual variation in population size to be better explained by the spline-based models. Small sample sizes of radiocollared panthers negatively impact the precision of this model. This issue is prevalent when working with small, isolated, and endangered populations. Nonetheless, the coefficient of variation (CV) for our model was 28%, which is slightly better than that obtained by McClintock et al. (CV = 29%; 2015) and comparable to the precision achieved by Sollmann et al. [38], the only other published study to date that has attempted to estimate the population density of panthers in South Florida.

The model averaged confidence intervals (Fig. A6.1) were large; in later years of the model, the upper bounds exceeded reasonable population estimates that could likely be supported within the breeding range of South Florida (e.g., 821 panthers in 2018). These elevated upper bounds are likely related to the small sample of radiocollared individuals, the overall low probability of a panther motor vehicle mortality in our study, and no (biologically informed) prior constraints being imposed on the population size. Given these issues, we recommend caution when interpreting these upper bounds. The most informative outputs of this model are the lower bounds for the panther population size (**Main Text Fig. 5**), which indicate the population may never have exceeded 235 individuals from 2000–2018.

As expected, the lower bounds of our model exceed the minimum number alive counts [39,40] (**Main Text Fig. 5**).

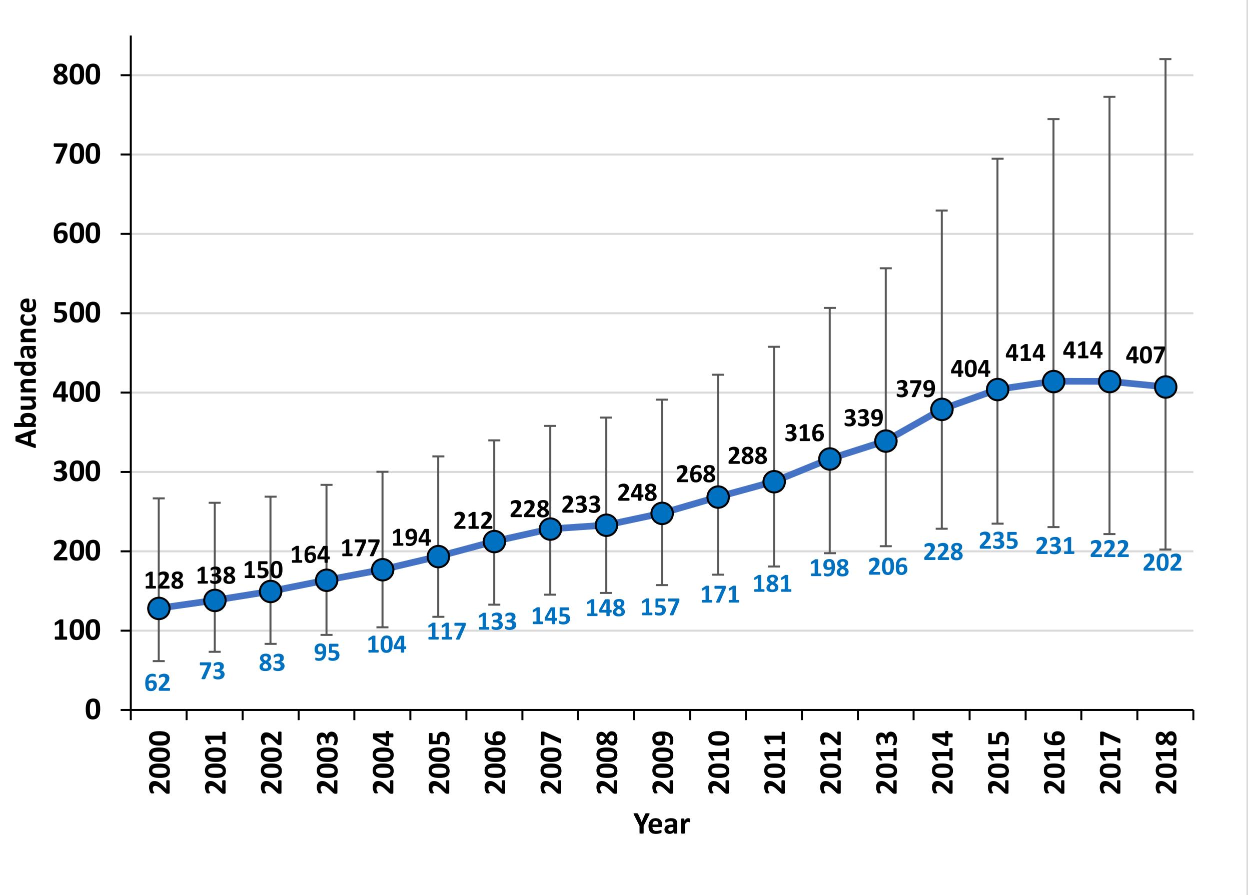

Figure A6.1 Estimates of the range-wide population size of adult and subadult Florida panthers from 2000–2018 using the motor vehicle mortality (MVM) model of McClintock et al. [37]. Model-averaged abundance estimates (black text) are presented with 95% confidence intervals. The lower 95% confidence interval values (blue text) were used for **Fig. 5** in the main text**.**

Supplemental Information Appendix 7

Table A7.1. Comparisons of estimates of genetic structure via F_{st} between Western puma populations and Florida panthers grouped by ancestry.

	Canonical	Admixed	Texas	Colorado	Idaho	North Dakota	South Dakota
Canonical	0.000						
Admixed	0.071	0.000					
Texas	0.255	0.105	0.000				
Colorado	0.278	0.140	0.069	0.000			
Idaho	0.265	0.127	0.076	0.039	0.000		
North Dakota	0.316	0.169	0.117	0.072	0.060	0.000	
South Dakota	0.272	0.139	0.082	0.048	0.053	0.072	0.000

Table A7.2. Comparisons of estimates of genetic structure via F_{st} between Western puma populations and Florida panthers grouped by cohorts born pre- post-genetic rescue.

	Pre1	Pre2	Post1	Post2	Post3	Texas	Colorado	Idaho	North Dakota	South Dakota
Pre1	0.000									
Pre2	0.015	0.000								
Post1	0.025	0.031	0.000							
Post2	0.048	0.054	0.009	0.000						
Post3	0.051	0.054	0.016	0.005	0.000					
Texas	0.178	0.215	0.120	0.106	0.125	0.000				
Colorado	0.195	0.236	0.153	0.145	0.161	0.069	0.000			
Idaho	0.182	0.224	0.140	0.131	0.146	0.076	0.039	0.000		
North Dakota	0.232	0.275	0.186	0.171	0.185	0.117	0.072	0.060	0.000	
South Dakota	0.201	0.234	0.153	0.141	0.153	0.082	0.048	0.053	0.072	0.000

Literature Cited

1. Taberlet, P. *et al.* Reliable genotyping of samples with very low DNA quantities using PCR. *Nucleic Acids Res.* **24**, 3189-3194 (1996).
2. McKelvey, K. S. & Schwartz, M. K. DROPOUT: a program to identify problem loci and samples for noninvasive genetic samples in a capture-mark-recapture framework. *Mol. Ecol. Notes* **5**, 716-718 (2005).
3. Weir, B. S. & Cockerham, C. C. Estimating F-Statistics for the Analysis of Population Structure. *Evolution* **38**, 1358-1370 (1984).
4. Guo, S. W. & Thompson, E. A. Performing the exact test of Hardy-Weinberg proportion for multiple alleles. *Biometrics* **48**, 361-372 (1992).
5. Raymond, M. & Rousset, F. GENEPOP (Version 1.2): population genetics software for exact tests and ecumenicism. *J. Hered.* **86**, 248-249 (1995).
6. Rousset, F. Genepop'007: a complete re-implementation of the genepop software for Windows and Linux. *Molecular Ecology Resources* **8**, 103-106 (2008).
7. Sokal, R. R. & Rohlf, F. J. *Biometry*. Third edn, (W. H. Freeman, 1995).
8. Van Oosterhout, C., Hutchinson, W. F., Wills, D. P. M. & Shipley, P. MICRO-CHECKER: software for identifying and correcting genotyping errors in microsatellite data. *Molecular Ecology Resources* **4**, 535-538 (2004).
9. Chakraborty, R., Andrade, M. d., Daiger, S. & Budowle, B. Apparent heterozygote deficiencies observed in DNA typing data and their implications in forensic applications. *Ann. Hum. Genet.* **56**, 45-57 (1992).
10. Johnson, W. E. *et al.* Genetic restoration of the Florida panther. *Science* **329**, 1641-1645 (2010).
11. Roelke, M. E., Martenson, J. S. & O'Brien, S. J. The consequences of demographic reduction and genetic depletion in the endangered Florida panther. *Curr. Biol.* **3**, 340-349 (1993).
12. Culver, M., Hedrick, P. W., Murphy, K., O'Brien, S. & Hornocker, M. G. Estimation of the bottleneck size in Florida panthers. *Anim. Conserv.* **11**, 104-110 (2008).
13. Frankham, R., Ballou, J. D. & Briscoe, D. A. *Introduction to Conservation Genetics*. (Cambridge University Press, Cambridge, UK, 2002).
14. Shirk, A., Wallin, D., Cushman, S., Rice, C. & Warheit, K. Inferring landscape effects on gene flow: a new model selection framework. *Mol. Ecol.* **19**, 3603-3619 (2010).
15. Pritchard, J. K., Stephens, M. & Donnelly, P. Inference of population structure using multilocus genotype data. *Genetics* **155**, 945-959 (2000).
16. Evanno, G., Regnaut, S. & Goudet, J. Detecting the number of clusters of individuals using the software STRUCTURE: a simulation study. *Mol. Ecol.* **14**, 2611-2620 (2005).
17. Earl, D. A. & vonHoldt, B. M. STRUCTURE HARVESTER: a website and program for visualizing STRUCTURE output and implementing the Evanno method. *Conservation Genetics Resources* **4**, 359-361 (2012).
18. Hostetler, J. A. *et al.* Genetic introgression and the survival of Florida panther kittens. *Biol. Conserv.* **143**, 2789-2796 (2010).
19. Zimmer, G. K. Animal release in Everglades National Park. *National Parks Magazine* **40**, 22-23 (1966).
20. Ochoa, A., Onorato, D. P., Fitak, R. R., Roelke-Parker, M. E. & Culver, M. Evolutionary and functional mitogenomics associated with the genetic restoration of the Florida Panther. *J. Hered.* **108**, 449-455 (2017).

21. Pike, N. Using false discovery rates for multiple comparisons in ecology and evolution. *Methods in Ecology and Evolution* **2**, 278-282 (2011).
22. R: A language and environment for statistical computing (R Foundation for Statistical Computing, Vienna, Austria, 2022).
23. Brooks, M. E. *et al.* glmmTMB balances speed and flexibility among packages for zero-inflated generalized linear mixed modeling. *The R Journal* **9**, 378-400 (2017).
24. Lenth, R. emmeans: estimated marginal means, aka least-squares means. . *R package version 1.7.5* (2022).
25. Hartig, F. DHARMa: Residual diagnostics for hierarchical (multi-level / mixed) regression models. *R package version 0.4.5* (2022).
26. Penfold, L. M. *et al.* Long-term evaluation of male Florida panther (*Puma concolor coryi*) reproductive parameters following genetic introgression. *J. Mammal.* **103**, 835-844 (2022).
27. Peakall, R. & Smouse, P. E. GENALEX 6: genetic analysis in Excel. Population genetic software for teaching and research. *Molecular Ecology Resources* **6**, 288-295 (2006).
28. Peakall, R. & Smouse, P. E. GenAlEx 6.5: genetic analysis in Excel. Population genetic software for teaching and research—an update. *Bioinformatics* **28**, 2537-2539 (2012).
29. Kalinowski, S. T. HP-RARE 1.0: a computer program for performing rarefaction on measures of allelic richness. *Mol. Ecol. Notes* **5**, 187-189 (2005).
30. Grueber, C. E., Wallis, G. P. & Jamieson, I. G. Heterozygosity–fitness correlations and their relevance to studies on inbreeding depression in threatened species. *Mol. Ecol.* **17**, 3978-3984 (2008).
31. Aparicio, J. M., Ortega, J. & Cordera, P. J. What should we weigh to estimate heterozygosity, alleles or loci? *Mol. Ecol.* **15**, 4659-4665 (2006).
32. Alho, J. S., Välimäki, K. & Merilä, J. Rhh: an R extension for estimating multilocus heterozygosity and heterozygosity-heterozygosity correlation. *Molecular Ecology Resources* **10**, 720-722 (2010).
33. van de Kerk, M., Onorato, D. P., Hostetler, J. A., Bolker, B. M. & Oli, M. K. Dynamics, persistence, and genetic management of the endangered Florida panther population. *Wildl. Monogr.* **203**, 3-35 (2019).
34. Ferrer, E. S., García-Navas, V., Sanz, J. J. & Ortego, J. The strength of the association between heterozygosity and probability of interannual local recruitment increases with environmental harshness in blue tits. *Ecology and Evolution* **6**, 8857-8869 (2016).
35. Harrell, F. E., Jr. *Regression modeling strategies: with applications to linear models, logistic regression, and survival analysis*. 572 (Springer Science, 2001).
36. Harrell, F. E., Jr. rms: regression modeling strategies. *R package version 6.3-0* (2022).
37. McClintock, B. T., Onorato, D. P. & Martin, J. Endangered Florida panther population size determined from public reports of motor vehicle collision mortalities. *J. Appl. Ecol.* **52**, 893-901 (2015).
38. Sollmann, R. *et al.* Using multiple data sources provides density estimates for endangered Florida panther. *J. Appl. Ecol.* **50**, 961-968 (2013).
39. McBride, R. & McBride, C. Florida panther annual count 2015. 34 (Rancher's Supply Inc., Ochopee, Florida, 2015).
40. McBride, R. T., McBride, R. T., McBride, R. M. & McBride, C. E. Counting pumas by categorizing physical evidence. *Southeast. Nat.* **7**, 381-400 (2008).
