# Supplementary material for: Multi-generational benefits of genetic rescue
Source: Sci Rep. 2024 Jul 30;14:17519. doi: 10.1038/s41598-024-67033-6 (PMC11289468; doi:10.1038/s41598-024-67033-6)
Supplement: Supplementary file 2 — Supplementary Legends. [file 41598_2024_67033_MOESM2_ESM.docx]

**Extended Data Fig. 1.** Results from STRUCTURE clustering analysis for Florida panthers with a *K* = 2, where the x-axis represents individual panthers in cohorts **a**) Pre1 (<1986), **b**) Pre2 (1986–1995), **c**) Post1 (1996–2005), **d**) Post2 (2006–2015), **e**) Post3 (2016–2020). Yellow indicates allele combinations associated with the canonical panthers and red indicates allele combinations associated with the admixture resulting from genetic rescue. The y-axis represents the percentage ancestry (*q*-values) for each ancestral group within each panther. The first 7 individuals in panel **c** are Texas female pumas released in 1995 (TX102 did not amplify); admixed bars for the Texas females are stippled red for clarity. Only values from panthers sampled as adults and subadults are included in this figure (n = 547). For the complete dataset including kittens, see *SI Appendix 2, Fig A2.1*.

**Extended Data Fig. 2.** Estimates of genetic effective population size (N_e_) for Florida panthers assessed via ancestry and cohorts of panthers born pre- and post-genetic rescue using genotype data from 16 microsatellite loci. Estimates of N_e_ for Western populations, including Texas, are presented for comparative purposes. Note the low N_e_ for North Dakota puma, reflective of a founder effect from recent recolonization of that portion of puma range [ref. ^74^](#_ENREF_74). The 95% parametric confidence intervals are presented with each bar. The upper 95% confidence intervals for Idaho (199) and South Dakota (1041) are not depicted on the figure to preserve a useful scale for N_e_ comparisons among populations.

**Extended Data Fig. 3.** Results from STRUCTURE clustering analysis for adult and subadult Florida panthers (n = 547) with a *K* = 3, where the x-axis represents individual panthers in cohorts **a**) Pre1 (<1986), **b**) Pre2 (1986–1995), **c**) Post1 (1996–2005),**d**) Post2 (2006–2015), **e**) Post3 (2016–2020). Frame **f** contains a sample of 49 Texas pumas, including 7 of the females released into South Florida in 1995, along with samples from Western populations in Colorado (n = 23) , Idaho (n = 23), North Dakota (n = 22) and South Dakota (n = 26). See *SI Appendix 2.* Yellow indicates allele combinations associated with the canonical panthers, red indicates allele combinations mainly associated with the admixture resulting from genetic rescue in panthers, and purple represents non-Florida allele combinations typically associated with Western pumas and the historic Everglades clade of panthers (see Roelke et al. ^12^).
