# Supplementary figures and images for: Multi-generational benefits of genetic rescue
Source: Sci Rep. 2024 Jul 30;14:17519. doi: 10.1038/s41598-024-67033-6 (PMC11289468; doi:10.1038/s41598-024-67033-6)

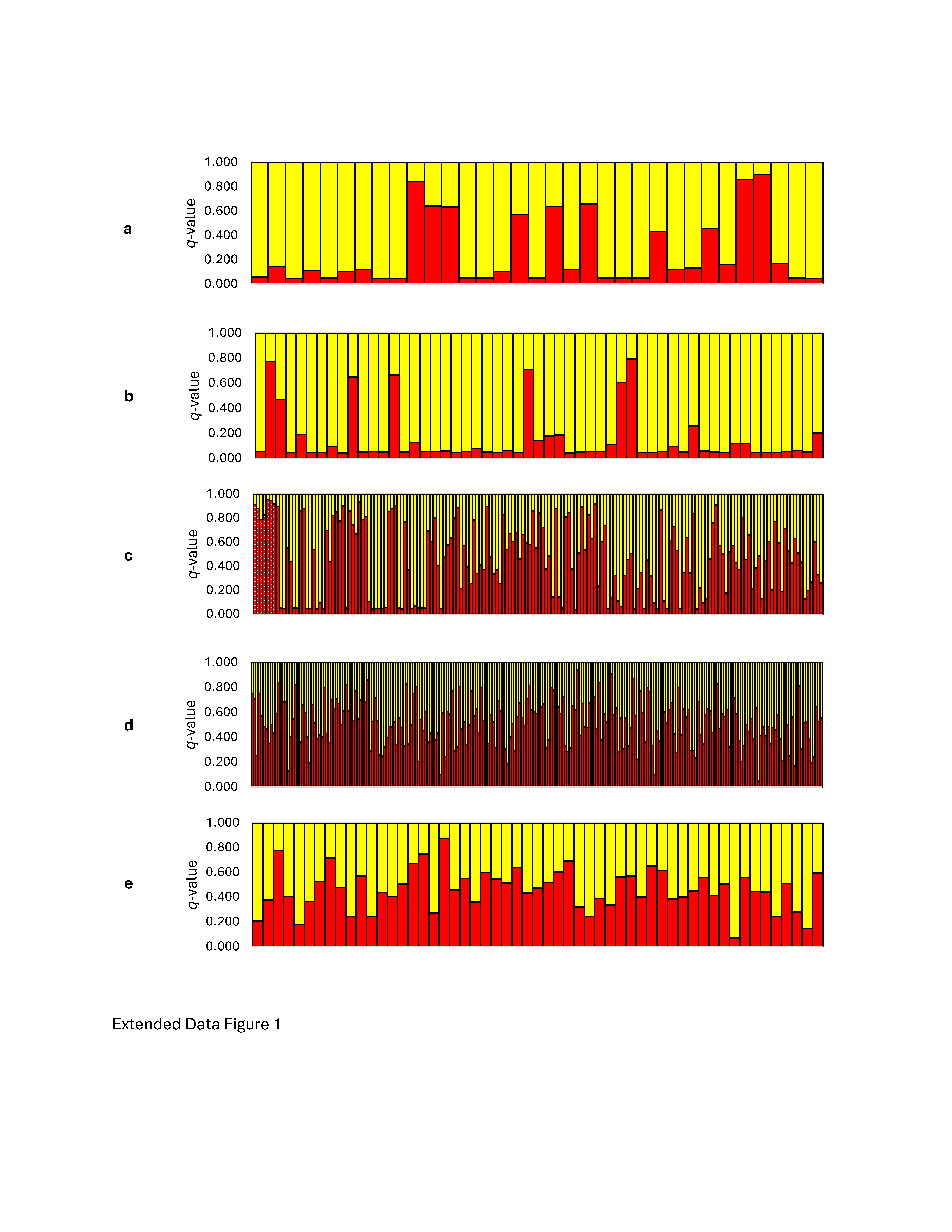

Supplement: Supplementary file 3 — Supplementary Figure 1. [file 41598_2024_67033_MOESM3_ESM.tiff]

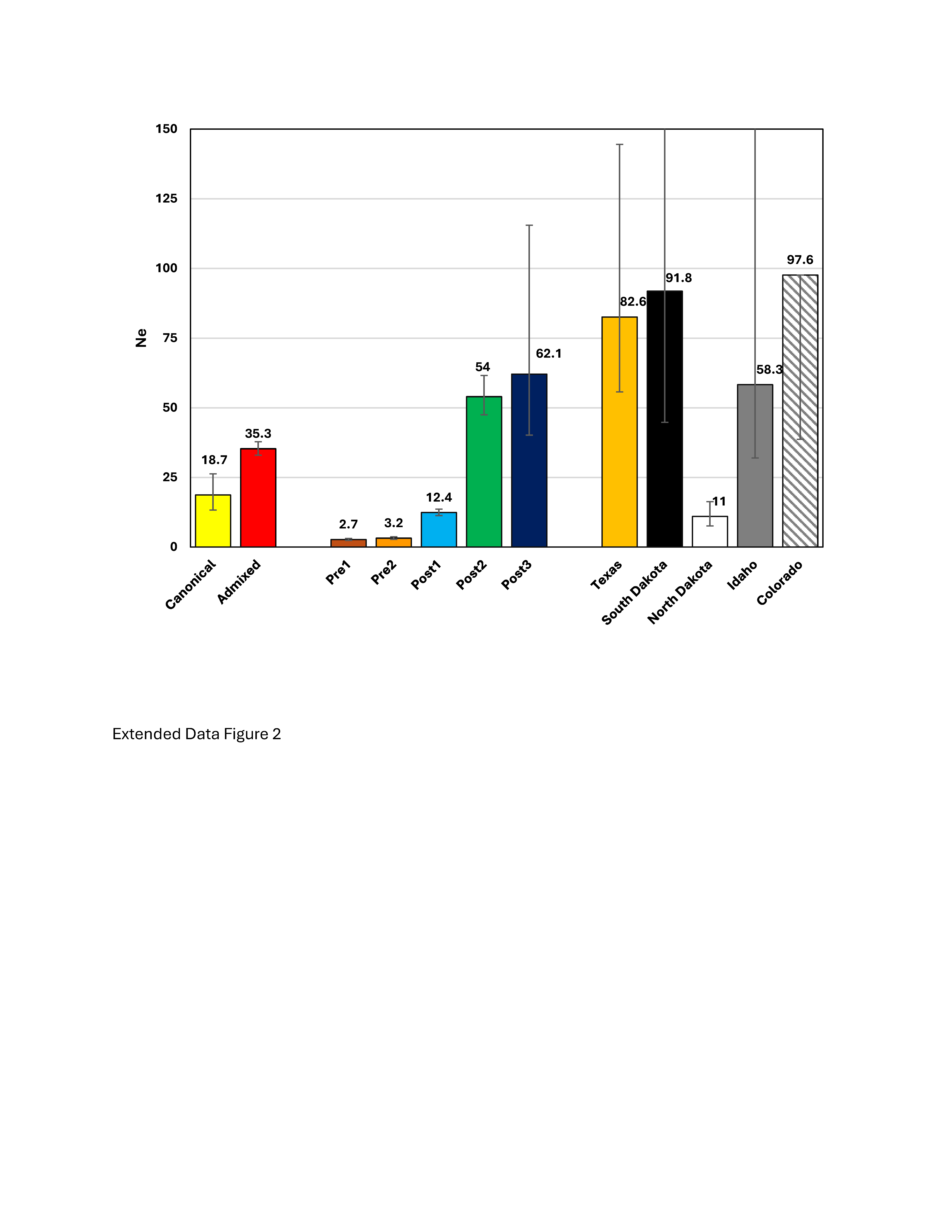

Supplement: Supplementary file 4 — Supplementary Figure 2. [file 41598_2024_67033_MOESM4_ESM.tiff]

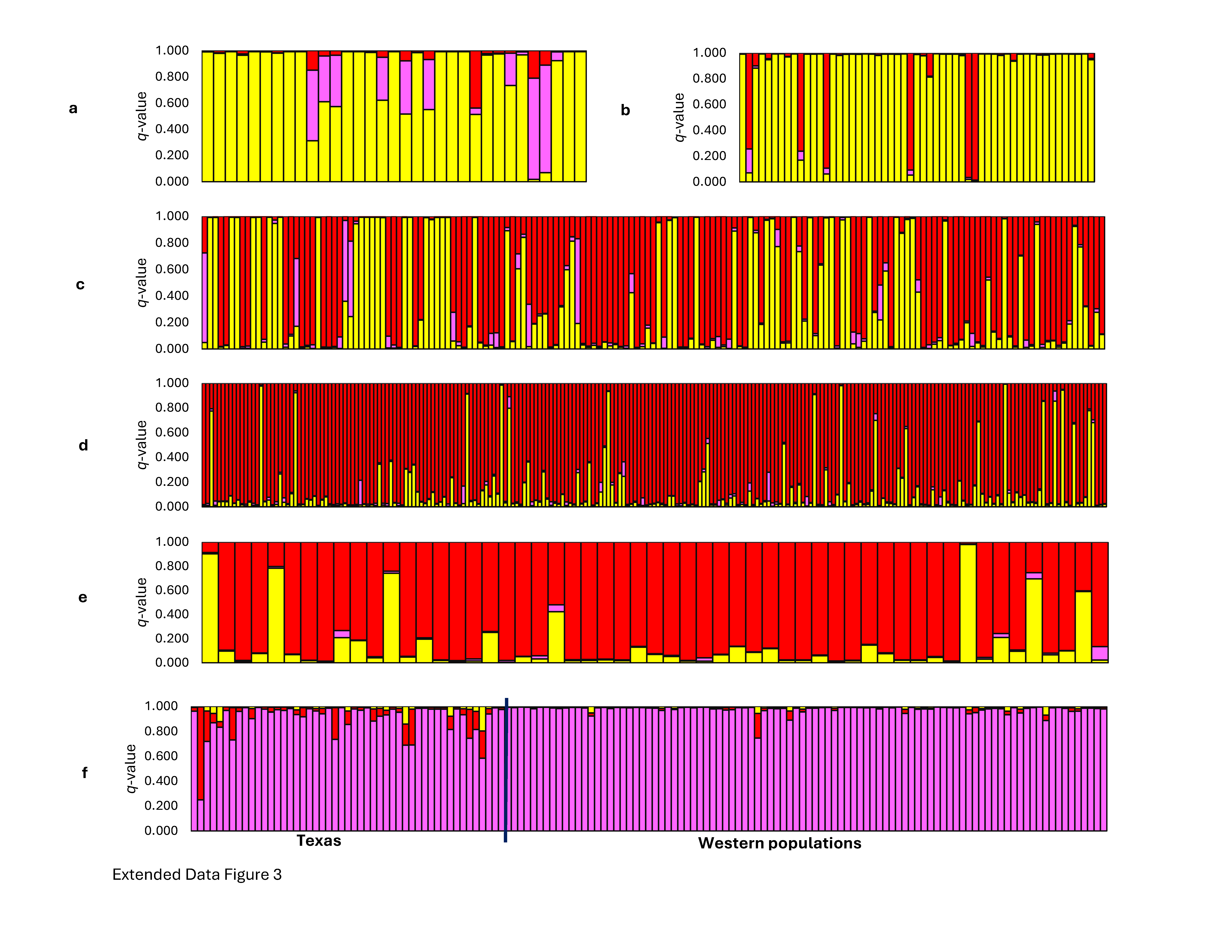

Supplement: Supplementary file 5 — Supplementary Figure 3. [file 41598_2024_67033_MOESM5_ESM.tiff]
